# Oncogenic BRAF, unrestrained by TGFβ-receptor signalling, drives right-sided colonic tumorigenesis

**DOI:** 10.1038/s41467-021-23717-5

**Published:** 2021-06-08

**Authors:** Joshua D. G. Leach, Nikola Vlahov, Petros Tsantoulis, Rachel A. Ridgway, Dustin J. Flanagan, Kathryn Gilroy, Nathalie Sphyris, Ester G. Vázquez, David F. Vincent, William J. Faller, Michael C. Hodder, Alexander Raven, Sigrid Fey, Arafath K. Najumudeen, Douglas Strathdee, Colin Nixon, Mark Hughes, William Clark, Robin Shaw, S:CORT consortium, Tim Maughan, Manuel Salto-Tellez, Philip Quirke, Viktor Koelzer, Philip Dunne, Andrew Beggs, Peter Campbell, Francesca Buffa, Chris Holmes, Rick Kaplan, Louise Brown, Mark Lawler, Joshua Hordern, Ian Tomlinson, Simon Leedham, Dion Morton, Sander R. van Hooff, David J. Huels, Jan Paul Medema, Simon T. Barry, Margaret C. Frame, Asier Unciti-Broceta, Simon J. Leedham, Gareth J. Inman, Rene Jackstadt, Barry J. Thompson, Andrew D. Campbell, Sabine Tejpar, Owen J. Sansom

**Affiliations:** 1 Cancer Research UK Beatson Institute, Glasgow, UK; 2 Institute of Cancer Sciences, University of Glasgow, Glasgow, UK; 3 Department of Medical Specialties, Faculty of Medicine, University of Geneva, Geneva, Switzerland; 4 Department of Oncology, Hôpitaux Universitaires de Genève, Geneva, Switzerland; 5 Gastrointestinal Stem Cell Biology Lab, Wellcome Trust Centre for Human Genetics, University of Oxford, Oxford, UK; 6 Division of Oncogenomics, Netherlands Cancer Institute, Amsterdam, The Netherlands; 7 Laboratory for Experimental Oncology and Radiobiology (LEXOR), Center for Experimental Molecular Medicine (CEMM) and Cancer Center Amsterdam, Academic Medical Center, Amsterdam, The Netherlands; 8 Oncode Institute, Academic Medical Center, Amsterdam, The Netherlands; 9 Bioscience, Early Oncology, AstraZeneca, Cambridge, UK; 10 Cancer Research UK Edinburgh Centre, MRC Institute of Genetics and Molecular Medicine, University of Edinburgh, Edinburgh, UK; 11 EMBL Australia, John Curtin School of Medical Research, The Australian National University, Canberra, ACT Australia; 12 Molecular Digestive Oncology, Department of Oncology, University of Leuven, Leuven, Belgium; 13 University of Oxford, Oxford, UK; 14 Queen’s University Belfast, Belfast, UK; 15 University of Leeds, Leeds, UK; 16 University of Bern, Bern, Switzerland; 17 University of Birmingham, Birmingham, UK; 18 Wellcome Sanger Institute, Hinxton, UK; 19 University College London, London, UK; 20 University of Edinburgh, Edinburgh, UK

**Keywords:** Cancer models, Cancer stem cells

## Abstract

Right-sided (proximal) colorectal cancer (CRC) has a poor prognosis and a distinct mutational profile, characterized by oncogenic *BRAF* mutations and aberrations in mismatch repair and TGFβ signalling. Here, we describe a mouse model of right-sided colon cancer driven by oncogenic BRAF and loss of epithelial TGFβ-receptor signalling. The proximal colonic tumours that develop in this model exhibit a foetal-like progenitor phenotype (*Ly6a/Sca1*^+^) and, importantly, lack expression of *Lgr5* and its associated intestinal stem cell signature. These features are recapitulated in human *BRAF*-mutant, right-sided CRCs and represent fundamental differences between left- and right-sided disease. Microbial-driven inflammation supports the initiation and progression of these tumours with foetal-like characteristics, consistent with their predilection for the microbe-rich right colon and their antibiotic sensitivity. While MAPK-pathway activating mutations drive this foetal-like signature via ERK-dependent activation of the transcriptional coactivator YAP, the same foetal-like transcriptional programs are also initiated by inflammation in a MAPK-independent manner. Importantly, in both contexts, epithelial TGFβ-receptor signalling is instrumental in suppressing the tumorigenic potential of these foetal-like progenitor cells.

## Abstract (web summary)

Right-sided colorectal cancer (rCRC) has a different mutational spectrum to the left-sided counterpart. Here the authors develop a mouse model of rCRC that recapitulates human BRAF-mutant rCRC and show that loss of TGFβ-receptor signalling and inflammation induce the development of colonic tumours with a foetal-like phenotype.

## Introduction

Colorectal cancer (CRC)—a leading cause of cancer-related mortality worldwide—is a heterogeneous group of neoplasms arising from the epithelium lining the large intestine/colon. In recent years, the anatomical location/sidedness of colorectal tumours has emerged as an important determinant of disease progression, response to systemic therapy, and clinical outcome. Indeed, colorectal tumours that arise proximal (right) or distal (left) to the splenic flexure manifest profound differences in epidemiology, histopathogenesis, and molecular landscapes^1–3^.

Left-sided CRCs develop from benign adenomas through the conventional adenoma-carcinoma pathway typically entailing aberrant activation of Wnt signalling, most prominently via biallelic inactivation of the tumour suppressor *APC*, or activating mutations in *CTNNB1*, both of which result in the stabilization and nuclear translocation of β-catenin. Progression of premalignant tubular, villous, or tubulovillous adenomas to adenocarcinoma is underpinned by accumulation of mutations in oncogenes and tumour suppressor genes, such as *KRAS*, *PIK3CA*, *TP53*, and *SMAD4*, as well as chromosomal instability^4,5^. Right-sided CRCs (rCRCs) are thought to arise through an alternative serrated neoplasia pathway so-called because the precursor lesions—namely, hyperplastic polyps, sessile serrated adenomas/lesions (SSAs), and traditional serrated adenomas (TSAs)—are distinguished by a distinctive saw-tooth crypt morphology caused by marked infolding of the upper crypt epithelium^6–8^. SSAs, in particular, carry a significant risk for malignant progression^6–8^. In addition to the saw-tooth crypt architecture seen in longitudinal sections, right-sided tumours often exhibit other common histopathological features, including a flat/sessile morphology, and a mucinous histology, as well as a propensity towards aggressive local invasion with peritoneal metastases/carcinomatosis^9–13^. rCRCs carry a worse prognosis than their left-sided counterparts and are characterised by microsatellite instability (MSI) and aberrations in MAPK, TGFβ, and mismatch repair pathways^9–11^.

Oncogenic mutation of *BRAF*, resulting in a valine-to-glutamate substitution at residue 600 of the BRAF serine-threonine kinase (BRAF^V600E^), has been identified as a key initiating event in a sizeable subset of serrated premalignant lesions and rCRCs^14,15^. Independent of upstream RAS signalling, the BRAF^V600E^ oncoprotein causes constitutive activation of the MAPK (RAS-RAF-MEK-ERK) signalling cascade, which plays key roles in cell survival, proliferation, differentiation, senescence, and apoptosis. Previous attempts to model rCRCs, utilising *Braf*^*V600E*^ alone, resulted primarily in small intestinal tumours displaying Wnt-pathway activation^16–18^. However, these findings are discordant with data from patients harbouring *BRAF*^*V600E*^right-sided colonic tumours that notably lack nuclear β-catenin positivity^19^. In respect of this apparent lack of Wnt-pathway activation, recent literature has implicated the Hippo-pathway effectors YAP/TAZ in the reprogramming and mobilization of foetal-like, *Lgr5*^−^ epithelial populations during the early stages of colitis-associated regeneration^20^. In this context, *Lgr5*^+^ intestinal stem cell (ISC) signatures are suppressed with a concomitant increase in the expression of foetal intestinal markers, such as *Ly6a*/*Sca1* and *Anxa1*, amongst others^21,22^. Similarly, YAP/TAZ signalling drives the transient foetal reprogramming that occurs during irradiation-induced regeneration, underpinned by suppression of *Lgr5*^+^ ISCs and attenuation of Wnt signalling^23^. Finally, this YAP/TAZ regenerative signalling has also been linked to the progression of APC-deficient foci to adenomas in the murine small intestine^23^. As chronic inflammation, and tissue injury and repair are associated with an elevated risk of CRC, it remains to be seen whether this YAP/TAZ-dependent, foetal-like, *Lgr5*^−^ regenerative state plays a role in the development of right-sided colonic tumours lacking Wnt-pathway aberrations.

To date, the lack of tractable tumour models that faithfully recapitulate the topography and molecular landscapes of oncogenic BRAF-driven rCRCs has hampered efforts to decipher the underlying molecular mechanisms and develop targeted therapeutics. Here, we describe a human-like mouse model of right-sided colon cancer driven by oncogenic BRAF and loss of epithelial TGFβ-receptor signalling that develops proximal colonic tumours with a foetal-like progenitor phenotype (*Ly6a/Sca1*^+^) and, importantly, lack expression of *Lgr5* and its associated intestinal stem cell signature. We further ascribe a role for microbial-driven inflammation in the initiation of these tumours, consistent with the long-held contention that intestinal microbiota are a key factor in CRC development and the pathogenesis of right-sided disease in particular^24,25^. Finally, whether induced directly by oncogenic BRAF or by inflammation, we show that the tumorigenic potential of these foetal-like progenitor populations is fundamentally restrained by epithelial TGFβ signalling.

## Results

### BRAF^V600E^ and TGFβ-receptor loss drive Wnt-low tumours in the right colon

**Fig. 1:**
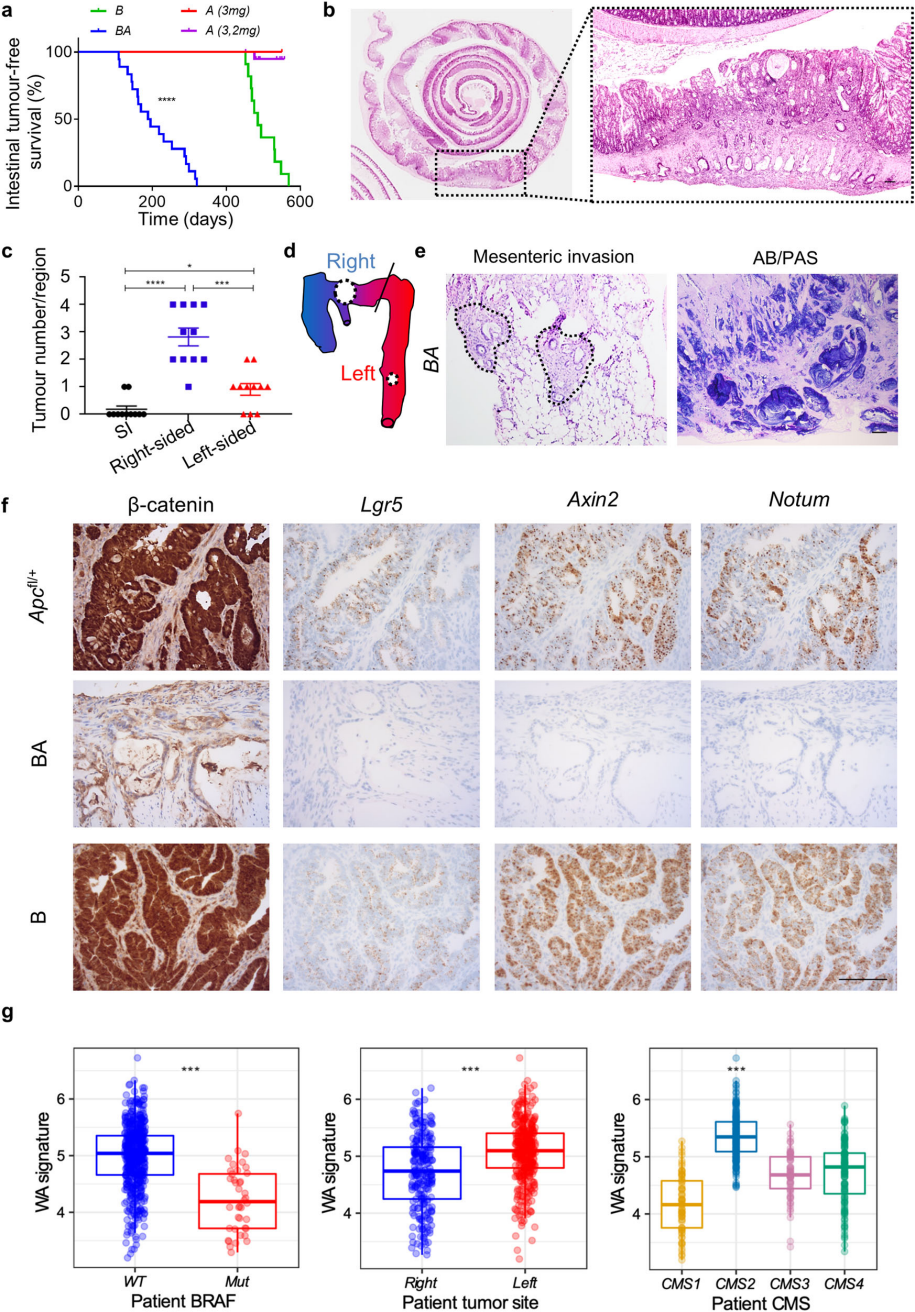
Epithelial-specific *Braf*^*V600E*^ mutation combines with loss of TGFβ-receptor signalling to drive right-sided tumorigenesis without Wnt-pathway activation. **a** Kaplan–Meier survival curves highlighting intestinal tumour-free survival (days after induction) of VillinCre^ER^; *Braf*^*V600E/+*^ (B; *n* = 11; 3 mg tamoxifen induction), VillinCre^ER^; *Alk5*^*fl/fl*^ *(*A; *n* = 3; 3 mg tamoxifen induction), and VillinCre^ER^; *Braf*^*V600E/+*^; *Alk5*^*fl/fl*^ (BA; *n* = 18; 2 mg tamoxifen induction) mice. A mice were censored at 550 days post induction (indicated by tick marks on the Kaplan–Meier curve). (****p = 1.835e−7 for comparison of B vs BA; Mantel-Cox log-rank test, two-tailed). **b** Representative H&E staining of BA mouse colon (*n* = 8) (Swiss-roll). Right, higher magnification of dashed area in left panel, highlighting the proximal (right-sided) colonic location of adenocarcinomas. Scale bar, 500 μm. **c** Intestinal tumour location scoring in BA mice (*n* = 11 mice). Mean ± s.e.m. (*p = 0.0194, ***p = 0.0003, ****p = 8.5e−6; Mann–Whitney *U*-test, two-tailed). **d** Schematic representing the tumour distribution in the intestinal tract of BA mice, with circle size proportional to the regional tumour number scored in (**c**). Blue, caecum, and proximal colon; Red, left colon (descending) to rectum; Diagonal line, splenic flexure. **e** Representative images of mesenteric invasion, with tumour epithelium highlighted with a dotted outline (H&E; left panel), and mucin staining (Alcian Blue/PAS; right panel) in primary tumour sections from BA mice (*n* = 5). Scale bar, 500 μm. **f** Representative tumour sections, from VillinCre^ER^; *Apc*^*fl/+*^, B, and BA mice (*n* = 5 mice per group), stained for β-catenin (immunohistochemistry) and the Wnt-pathway activation markers *Lgr5*, *Axin2*, and *Notum* (ISH). Scale bar, 100 μm. **g** Boxplots of the Wnt activation (WA) signature in WT vs *BRAF*-mutant CRCs from patients, right- vs left-sided CRCs, and across CMS subtypes. Median is shown with boxes which extend from the 25th to the 75th percentile and whiskers which extend an additional 1.5× the interquartile range. Left panel *n* = 579, middle *n* = 604 and right *n* = 467 independent samples (Left and middle panels: ***p < 0.001, *T*-test, two-tailed; Right panel: ***p < 2e−16, *T*-test, two tailed).

Given the strong association of rCRCs with mutations in *BRAF*, defects in TGFβ-receptor signalling, and epigenetic silencing of the mismatch repair gene *MLH1*^2^, we targeted these mutations to the murine intestinal tract. We used the tamoxifen-inducible intestinal epithelial cell-specific VillinCre^ER^ transgene to conditionally activate the *Braf*^*LSL-V600E*^ knock-in allele, or to delete the floxed alleles of the genes encoding the TGFβ-receptor TGFBR1/ALK5 or the mismatch repair protein MLH1 (*Tgfbr1/Alk5*^*fl/fl*^ or *Mlh1*^*fl/fl*^, respectively)^26–28^. VillinCre^ER^; *Alk5*^*fl/fl*^ mice (designated A) did not develop tumours as a result of the indicated induction regime (3 mg on one occasion), while a further cohort of 20 mice subject to a more stringent regime (3 mg, 2 mg on consecutive days) did not develop tumours by 475 days (Fig. 1a). In addition, VillinCre^ER^; *Mlh1*^*fl/fl*^ and VillinCre^ER^; *Braf*^*LSL-V600E/+*^ (designated B) mice developed tumours predominantly in the small intestine (Fig. 1a, Supplementary Fig. 1a, b).

We reasoned that TGFβ-receptor inactivation in human cancer—occurring as a result of defective mismatch repair (due to a microsatellite sequence in *TGFBR2*, missing from the mouse genome)^29^—might synergise with mutant BRAF to initiate right-sided colonic tumours. Indeed, VillinCre^ER^; *Braf*^*LSL-V600E/+*^; *Alk5*^*fl/fl*^ (designated BA) mice developed tumours rapidly compared with B mice, with median survival times of 192 and 485 days, respectively (Fig. 1a). Crucially, BA mice developed adenocarcinomas predominantly in the proximal colon, which is equivalent to the right colon in humans (Fig. 1b–d). Further recapitulating rCRC, BA tumours exhibited a flat/sessile morphology, mucinous differentiation, and a pronounced ability to invade the mesentery (Fig. 1b, e).

To ensure that the preferential right-sidedness of tumours in BA mice was not due to a regional variation in recombination efficiency along the length of the intestinal tract, we scored tdTomato-reporter expression in VillinCre^ER^; *Rosa26*^*LSL-tdTomato/+*^ mice following varying doses of tamoxifen. Indeed, at doses of 80 mg/kg, whilst emergent tumours were right-sided, the recombination efficiency was at its lowest in the colon and caecum compared with the small intestine, highlighting that the preferential right-sidedness of tumours in this model is a result of the specific genetic context (Supplementary Fig. 1c).

TGFβ growth-inhibitory signals are transduced through a hetero-tetrameric receptor complex comprised of TGFBR1/ALK5 and TGFBR2 homodimers. Given that mutations in *TGFBR2* are more common than in *TGFBR1* in human tumours^30^, we also generated VillinCre^ER^; *Braf*^*V600E/+*^; *Tgfbr2*^*fl/fl*^ mice^31^. These mice also developed primarily proximal colonic adenocarcinomas directly recapitulating the BA model (Supplementary Fig. 1d). Importantly, this shared phenotype between BA and VillinCre^ER^; *Braf*^*V600E/+*^; *Tgfbr2*^*fl/fl*^ mice ruled out the involvement of non-canonical receptor-specific TGFβ signalling in the suppression of right-sided tumorigenesis.

We next sought to determine whether BA tumours exhibited Wnt-pathway activation. Unlike APC-deficient (VillinCre^ER^; *Apc*^*fl/+*^) or B tumours, BA tumours did not show nuclear β-catenin accumulation (Fig. 1f). Furthermore, we detected diminished expression of select Wnt-target genes (*Lgr5*, *Axin2*, and *Notum*) and our previously reported Wnt-target gene signature^32^, confirming that these right-sided colonic tumours lack Wnt-pathway activation (Fig. 1f, Supplementary Fig. 2). Using RNA-sequencing (RNA-seq) data from VillinCre^ER^; *Apc*^*fl/fl*^ intestinal tissues, harvested on day 4 post induction, we next derived a Wnt activation (WA) signature (Supplementary Fig. 3a), filtered to retain only epithelial genes that cluster close to *AXIN2* (a prominent transcriptional target of β-catenin-dependent Wnt signalling) in bulk human gene expression data^33–35^. Importantly, this signature was significantly downregulated in patient tumours with *BRAF*-mutant status compared with *BRAF* wild-type counterparts, right-sided vs left-sided location, and the CRC consensus molecular subtype (CMS) 1 (enriched for MSI and inflammatory/immune gene expression) vs CMS2 (enriched for genes associated with canonical Wnt signalling; Fig. 1g)^36^. Collectively, these findings demonstrate that although BRAF activation alone is not sufficient to drive tumour formation in the right colon, concurrent loss of epithelial TGFβ-receptor signalling cooperates in the genesis of proximal colonic tumours with histological features of human rCRCs and attenuated Wnt/β-catenin signalling.

### BA tumours exhibit foetal-like differentiation from early initiation

**Fig. 2:**
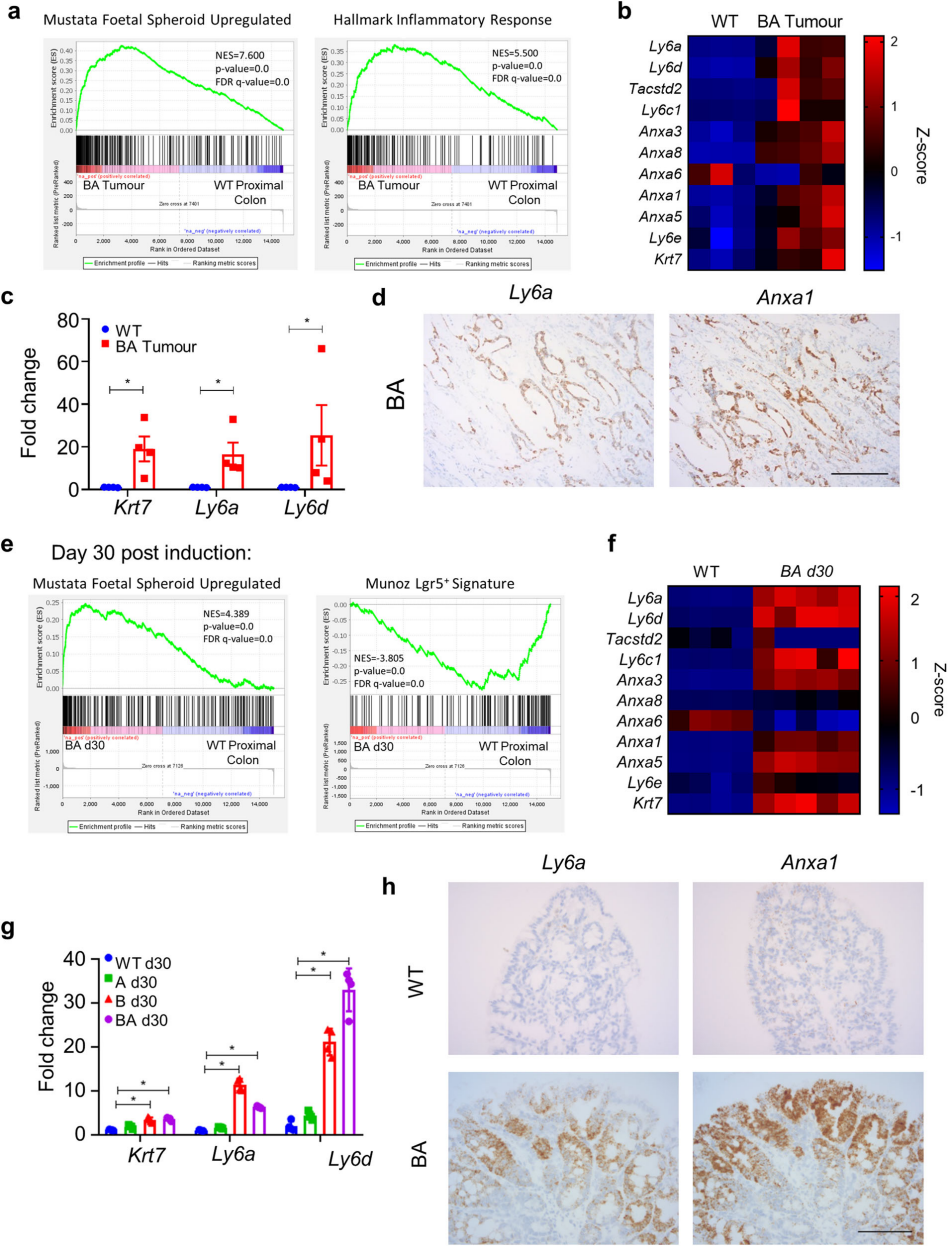
Wnt-low, right-sided tumours express a foetal-like signature, present from early initiation. **a** GSEA plots showing enrichment of foetal spheroid and HALLMARK inflammatory response signatures in endpoint BA colonic tumours (*n* = 4) vs WT proximal colon (*n* = 3). NES normalised enrichment score, FDR false discovery rate. (*p* = 0.0, represents *p* < 0.001 calculated empirically.) **b** Heatmap showing Z-score-transformed relative expression levels of selected foetal signature genes differentially expressed between endpoint BA intestinal tumours (*n* = 4) and WT proximal colonic tissue (*n* = 3). **c** qRT-PCR of indicated foetal markers in BA intestinal tumours vs WT proximal colon (*n* = 4 mice per group). Mean ± s.e.m. (*p = 0.0286, compared with WT; Mann–Whitney *U*-test, two-tailed). **d** Representative ISH of BA intestinal tumour epithelium stained for foetal markers *Ly6a* and *Anxa1* (*n* = 5 mice). Scale bar, 100 μm. **e** GSEA plots showing positive enrichment of the foetal spheroid signature (left panel) and negative enrichment of the *Lgr5*^+^ ISC signature (right panel) in BA proximal colonic tissue vs WT control tissue, 30 days post induction (*n* = 5 per group). NES normalised enrichment score, FDR false discovery rate. (*p* = 0.0, represents *p* < 0.001 calculated empirically.) **f** Heatmap showing Z-score-transformed relative expression levels of selected foetal signature genes in BA proximal colonic tissues (*n* = 5), compared with WT (*n* = 4), 30 days post induction. **g** qRT-PCR of indicated foetal markers in A, B, and BA vs WT proximal colonic tissues, 30 days post induction (*n* = 4 per group). Mean ± s.e.m. (*p = 0.0286, compared with WT; Mann–Whitney *U*-test, two-tailed). **h** Representative ISH of WT and BA proximal colonic tissue, stained for foetal markers *Ly6a* and *Anxa1*, 30 days post induction (*n* = 5 per group). Scale bar, 100 μm.

Due to the notable absence of *Lgr5*^+^ cells in BA tumours, we next ascertained whether these tumours recapitulated the *Lgr5*-independent, foetal spheroid signature^21^ associated with the regenerative response that ensues following ionizing radiation or dextran sulphate sodium salt (DSS) treatment^20,23^. RNA-seq and gene set enrichment analysis (GSEA) revealed a strong association of BA tumours with this foetal-like signature compared with VillinCre^ER^ (WT) controls (Fig. 2a–c, Supplementary Table 1). Of note, these foetal markers included *Ly6a* and *Anxa1*, two genes highly expressed during DSS-induced regeneration^20^. We also found enrichment for genes associated with an inflammatory response within BA tumours (Fig. 2a, right panel)^37^. Given that a number of foetal markers may be expressed by stromal cells, particularly the immune infiltrate, we confirmed epithelial cell-specific expression of select foetal markers (*Ly6a* and *Anxa1*) via in situ hybridization (ISH) (Fig. 2d). Interestingly, cytokeratin 7—a keratin not normally expressed in adult intestinal epithelium, but a component of the foetal gene program—is strongly expressed in BA colonic tumour epithelium (Supplementary Fig. 1e). Indeed, CK7 has been put forth as an independent marker of the serrated pathway in CRC^38^.

We next addressed whether the foetal-like gene signature emerges during tumorigenesis or whether it is present early on, from tumour initiation. For this, we harvested proximal colonic tissue from BA and WT mice, on day 30 (d30) post induction, and used RNA-seq to examine the expression of the same foetal markers at this early timepoint. Indeed, we found a significant enrichment of the foetal-like gene signature, as well as suppression of the *Lgr5*^+^ ISC signature^22^ (Fig. 2e–g, Supplementary Table 1) in d30 BA tissues relative to the WT proximal colon. Again, we confirmed epithelial cell-specific expression of *Ly6a* and *Anxa1* by ISH (Fig. 2h).

**Fig. 3:**
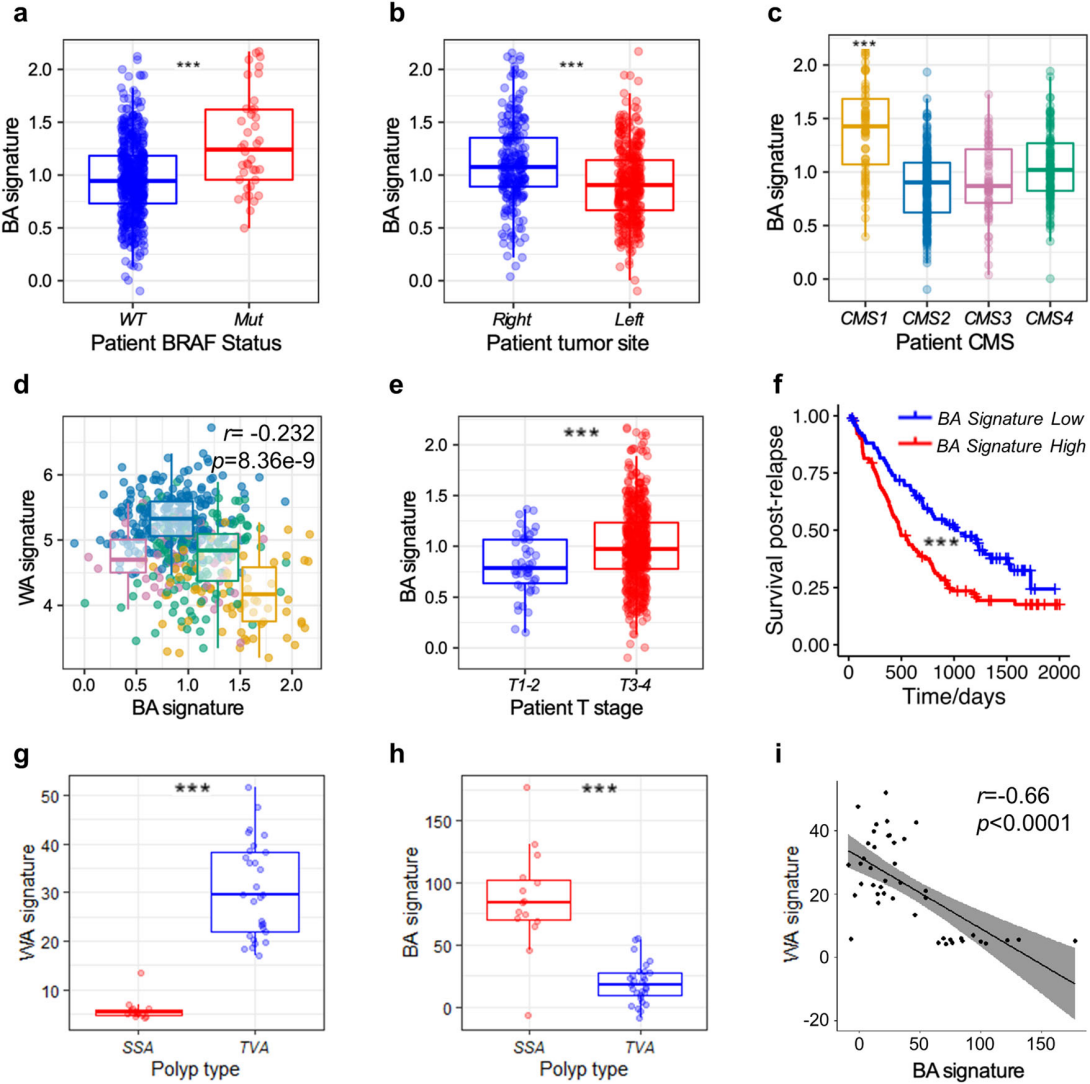
Poor-prognosis *BRAF*-mutant, right-sided patient CRCs are Wnt-low and express the BA signature from inception. **a** Boxplot showing expression of the BA signature in WT vs *BRAF*-mutant CRCs from patients (*n* = 579). Median is shown with boxes, which extend from the 25th to the 75th percentile and whiskers which extend an additional 1.5× the interquartile range (***p = 3.58e−5; T-test, two-tailed). **b** Boxplot showing expression of the BA signature of right- vs left-sided CRCs from patients (*n* = 604). Median is shown with boxes, which extend from the 25th to the 75th percentile and whiskers which extend an additional 1.5× the interquartile range (***p = 1.28e−9; T-test, two-tailed). **c** Boxplot showing expression of the BA signature across CMS subtypes (*n* = 461). Median is shown with boxes, which extend from the 25th to the 75th percentile and whiskers which extend an additional 1.5x the interquartile range (***p = 2.16e−13; T-test, two-tailed). **d** Scatter plot of WA and BA signature values from human tumours showing a negative correlation (Pearson’s *r* = −0.232, *p* = 8.36e−9, two-sided) (*n* = 467). CMS plots are overlain highlighting the differential expression of the signatures between CMS1 and CMS2 subtypes. CMS1 (MSI-high, immune): orange; CMS2 (canonical Wnt signalling): blue; CMS3 (metabolic dysregulation): pink; CMS4 (mesenchymal): green. Median is shown with boxes, which extend from the 25th to the 75th percentile and whiskers which extend an additional 1.5x the interquartile range. **e** Expression of the BA signature is higher in T3–4 human tumours than T1–2 (***p = 0.0002; T-test, two-tailed) (*n* = 604). Median is shown with boxes, which extend from the 25th to the 75th percentile and whiskers which extend an additional 1.5× the interquartile range. **f** Patients with BA signature-high tumours (red), defined as above the median value, have shorter survival after relapse compared with those harbouring BA signature-low tumours (blue) (***p = 0.00016; Wald test, two-tailed) (*n* = 197). **g** Expression of the WA signature is higher in tubulovillous adenomas (TVA) (*n* = 29) than sessile serrated adenomas (SSA) (*n* = 15) (***p = 1.538e−05; T-test, two-tailed). Median is shown with boxes, which extend from the 25th to the 75th percentile and whiskers which extend an additional 1.5× the interquartile range. **h** Expression of the BA signature is higher in sessile serrated adenomas (SSA) than tubulovillous adenomas (TVA) (***p = 1.498e−05; *T*-test, two-tailed). Median is shown with boxes, which extend from the 25th to the 75th percentile and whiskers which extend an additional 1.5× the interquartile range. **i** Higher values of the BA signature are significantly associated with lower expression values of the WA signature in pooled TVA/SSA human precancerous lesions (Pearson’s *r* = −0.66, *p* = 1.11e−06). The regression line is presented with 95% confidence intervals (shaded area).

Having demonstrated low Wnt-pathway activation in rCRCs and our BA model (Fig. 1f, g, Supplementary Fig. 2), we next generated an analogous epithelial-specific gene signature (designated BA) from our endpoint tumour and d30 BA RNA-seq data (Supplementary Fig. 3b). Using this BA signature to interrogate CRC-patient datasets^34,35^, we identified a significant correlation with *BRAF* mutation, right-sidedness, and the CMS1 subtype in CRC-patient tumours (Fig. 3a–c). We also found a significant inverse correlation between our BA and WA signatures and their associated CMS subtypes in patient samples as well as a positive correlation between our BA signature and the foetal spheroid signature of Mustata et al.^21^ (Fig. 3d, Supplementary Fig. 3c). Additionally, we observed a significant association between our BA signature and more advanced primary tumour stage (T3–4), consistent with the locally aggressive behaviour of our BA model (Fig. 3e). Accordingly, our BA signature also correlated with the shorter survival of relapsing patients and showed a trend towards an increased frequency of peritoneal disease upon relapse (Fig. 3f, Supplementary Fig. 3d), consistent with the pattern of metastatic spread in patients with rCRCs^12,13^.

We also observed a significant enrichment of the BA signature in sessile serrated adenomas/lesions (SSAs) compared with tubulovillous adenomas (TVAs), the precancerous lesions of CRCs associated with mutations in *BRAF* and Wnt-pathway components, respectively (Fig. 3g, h)^39^. Furthermore, we found a striking negative correlation between the WA and BA signatures in these same precancerous lesions implying a fundamental difference in their underlying biology from inception (Fig. 3i)^39^. These data support the notion that cells expressing the foetal-like BA signature are present from the early stages of human right-sided tumorigenesis, in similarity with our findings in the BA model where this signature was present from early tumorigenesis.

### BRAF^V600E^ promotes YAP-dependent foetal-like differentiation

**Fig. 4:**
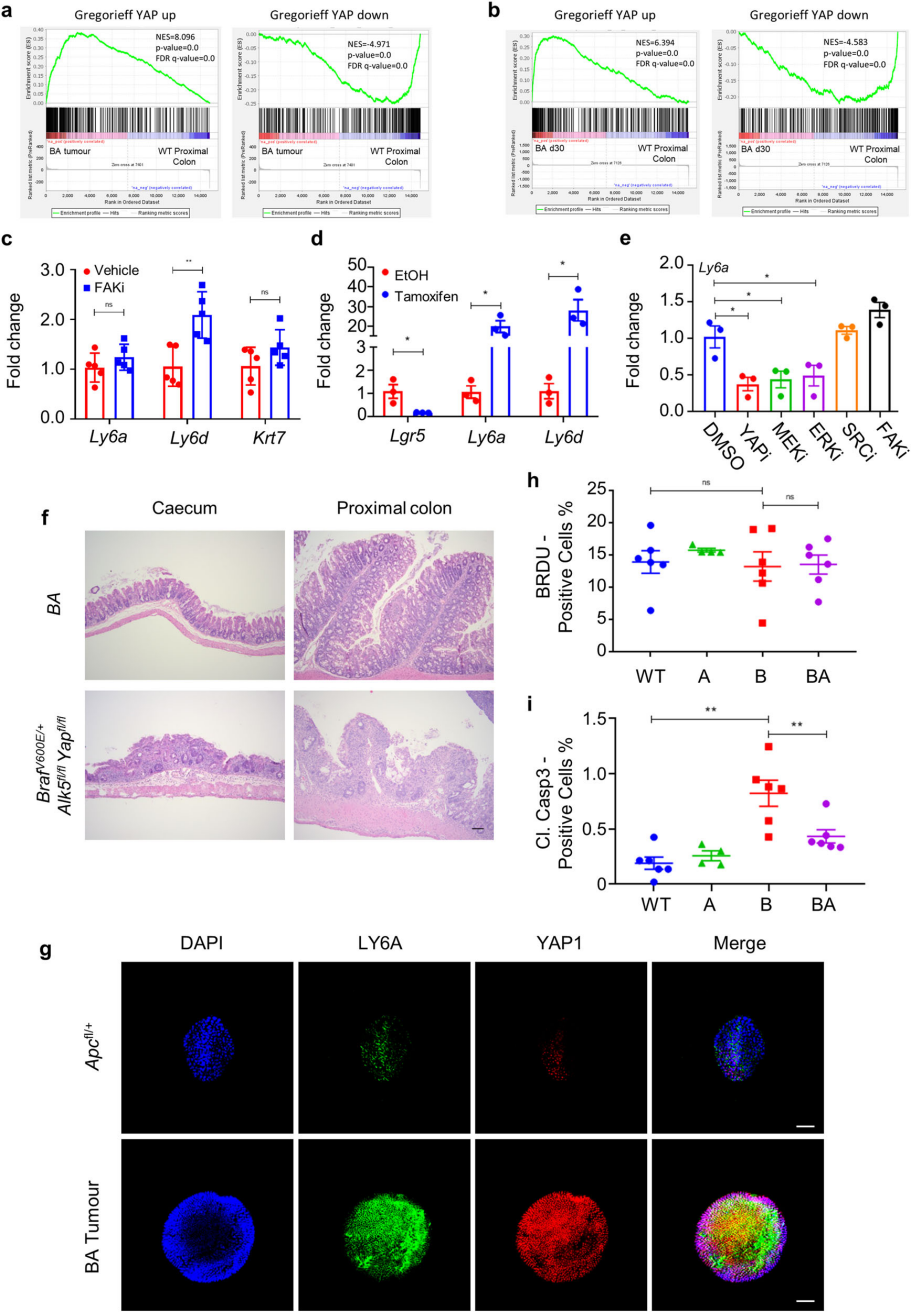
BRAF^V600E^ drives foetal-like differentiation via YAP activation, underpinning cell viability in the proximal colon. **a** GSEA plots showing enrichment of YAP up- and down-regulated gene signatures in endpoint BA tumours (*n* = 4) vs WT proximal colon (*n* = 3). NES normalised enrichment score, FDR false discovery rate. (*p* = 0.0, represents *p* < 0.001 calculated empirically.) **b** GSEA plots showing positive enrichment of YAP up- and down-regulated gene signatures in BA proximal colonic tissue vs WT control tissue, 30 days post induction (*n* = 5 per group). NES normalised enrichment score, FDR false discovery rate. (*p* = 0.0, represents *p* < 0.001 calculated empirically.) **c** qRT-PCR of indicated foetal markers in proximal colonic tissue from vehicle- and FAKi-(VS-4718)-treated BA mice, 30 days post induction (*n* = 5 mice per treatment). Mean ± s.e.m. (ns not significant; **p = 0.0079; Mann–Whitney *U*-test, two-tailed). **d** qRT-PCR of *Lgr5* and indicated foetal markers in BA organoids, induced in vitro with 4-hydroxytamoxifen and sampled 5 days post induction (*n* = 3 organoid lines from 3 separate uninduced BA mice). Fold change is shown relative to uninduced organoids (EtOH-treated). Mean ± s.e.m. (*p = 0.05; Mann–Whitney *U*-test, two-tailed). **e** qRT-PCR of *Ly6a* in BA organoids, induced in vitro with 4-hydroxytamoxifen and sampled 48 h post drug-treatment, 5 days post induction. Drugs were reconstituted in DMSO vehicle (0.1%) at the indicated final concentrations: verteporfin (YAPi; 3 μM), AZD6244 (MEKi; 100 nM), ERKi (100 nM), eCF506 (SRCi; 50 nM), and VS-4718 (FAKi; 1 μM). *n* = 3 organoid lines from 3 separate uninduced BA mice. Fold change is shown relative to DMSO-treated, induced organoids. Mean ± s.e.m. (*p = 0.05; Mann–Whitney *U*-test, two-tailed). **f** Representative H&E-staining of the proximal colon and caecum from *Braf*^V600E/+^ *Alk5*^fl/fl^ *Yap*^fl/fl^ mice 6 days post induction, highlighting severe epithelial loss and necrosis compared with BA controls (*n* = 5 mice per group). Scale bar, 500 μm. **g** Immunofluorescent staining of Matrigel ENRW-cultured BA and VillinCre; *Apc*^*fl/+*^ tumour spheroids for SCA1/LY6A (green) and YAP1 (red). Nuclei were counterstained with DAPI (blue). Merged images are shown on the right. Representative images of *n* = 3 biological replicates. Scale bar, 100 μm. **h** Image analysis scoring of the percentage of BrdU^+^ cells in the proximal colon epithelium of WT (*n* = 6), A (*n* = 4), B (n = 6), and BA (*n* = 6) mice, 30 days post induction. Mean ± s.e.m. (ns not significant; Mann–Whitney *U*-test, two-tailed). **i** Image analysis scoring of percentage of cleaved caspase 3^+^ cells in the proximal colon epithelium of WT (*n* = 6), A (*n* = 4), B (*n* = 6), and BA (*n* = 6) mice, 30 days post induction. Mean ± s.e.m. (**p = 0.0076, Mann–Whitney *U*-test, two-tailed).

Given the central role of the transcriptional coactivator YAP in foetal reprogramming during injury-induced regeneration^20,23^, we next examined our endpoint tumour and d30 RNA-seq data to determine whether YAP activity is associated with the foetal-like phenotype in our BA model. Utilising the YAP target-gene signature, generated by Gregorieff *et al*., we identified a strong correlation with YAP activation in both the endpoint tumour and d30 BA colonic tissues (Fig. 4a, b, Supplementary Table 1)^23^. Top-ranking genes in this YAP signature notably include foetal markers, such as *Ly6a* and *Anxa1*, as well as classic YAP targets such as *Ctgf*^23^.

We next used well-characterised and efficacious pharmacological agents to interrogate the signalling pathways eliciting YAP activation in our BA model. Previous studies have suggested that the activation of YAP, triggered upon remodelling of the extracellular matrix during DSS-induced colitis and regeneration, is dependent on FAK signalling^20^. However, we were unable to suppress the foetal program in vivo using the FAK-inhibitor VS-4718^40,41^ (FAKi), suggesting that mechanotransduction is not involved in the activation of YAP in our BA model (Fig. 4c).

Evaluation of intestinal FITC-dextran uptake in both B and BA mice revealed a significant intestinal barrier defect (Supplementary Fig. 4b), which was associated with pronounced proprial neutrophil infiltration in the caecum and proximal colon (Supplementary Fig. 4c). This locoregional inflammation, which co-localized with the formation of tumour foci, suggested a possible role for microenvironmental cues in triggering the foetal-like phenotype in our BA model. We therefore generated organoids from uninduced BA and B mice and induced gene recombination in vitro. Importantly, under these sterile culture conditions and in the absence of stromal influences, induction of mutant *Braf* alone was sufficient to activate the foetal-like program, accompanied by loss of *Lgr5* and upregulation of foetal markers (Fig. 4d, Supplementary Fig. 5a). In the organoid setting, induction of foetal marker expression was not affected by selective inhibitors of FAK (VS-4718)^40,41^ or SRC (eCF506)^42^, but it was significantly suppressed by inhibitors of MEK (AZD6244)^43,44^, ERK^45^, and YAP (verteporfin)^20^ (Fig. 4e), with efficacy of MEK and ERK inhibitors demonstrated by suppression of the canonical MAPK target *Dusp6* (Supplementary Fig. 5b). Notably, treatment of BA mice with the MEK-inhibitor AZD6244^43,44^ (MEKi) markedly suppressed the expression of foetal markers confirming the role of the MAPK pathway in the elaboration of the foetal-like phenotype in vivo (Supplementary Fig. 4a). Together, these results suggest that MAPK-activating mutations directly engage with YAP signalling and, consequently, induce foetal reprogramming in an epithelial cell-intrinsic, ERK-dependent manner. In this regard, the suppression of the foetal-like phenotype through YAP inhibition in vitro raised the possibility that the foetal-like differentiation program may be an adaptive survival mechanism directly initiated in *Braf*-mutant epithelium. To test the role of YAP in vivo using a genetics approach, we generated *VillinCre*^*ER*^*; Braf*^*V600E/+*^*; Alk5*^*fl/fl*^*; Yap*^*fl/fl*^ mice. Upon tamoxifen-induction, these mice rapidly developed weight loss and abdominal hunching, and had to be sampled on day 6 post induction due to these clinical signs. Histological evaluation of *VillinCre*^*ER*^*; Braf*^*V600E/+*^*; Alk5*^*fl/fl*^*; Yap*^*fl/fl*^ intestinal tissues confirmed the necessity of YAP for intestinal epithelial cell survival since, in its absence, we detected marked epithelial necrosis that was most pronounced in the proximal colon, although it was also evident throughout the rest of the intestine (Fig. 4f). Consistent with previous literature, tamoxifen-induced *VillinCre*^*ER*^*; Yap*^*fl/fl*^ mice showed no discernible phenotype^46^, confirming that YAP is dispensable for normal intestinal homeostasis.

We also observed that whilst *Braf* mutant organoids (B and BA) induced with tamoxifen in vitro grew in Matrigel, they could only be successfully passaged over the longer term in the presence of PGE2, a potent inflammatory mediator that has previously been shown to be a key component of the culture medium used to support the passage of foetal intestinal spheroids^47^. Endpoint tumour BA spheroids also exhibited this PGE2-dependency and maintained the same foetal-like phenotype in long-term culture as evidenced by LY6A expression and YAP1 nuclear localization (Fig. 4g). Given the locoregional inflammation in our BA model of right-sided colonic tumorigenesis, the requirement of exogenous PGE2 for long-term organoid passage is likely significant in light of the recently described requirement for pericryptal stromal production of PGE2 during post-injury wound healing^48^. This suggests that the combined inhibition of YAP and COX2, the enzyme that synthesizes PGE2, may hold therapeutic promise for the treatment of mutant BRAF-driven rCRCs.

Importantly, the ability of mutant BRAF alone to drive foetal-like differentiation demonstrated that TGFβ signalling does not suppress the foetal phenotype per se but, instead, independently restrains tumorigenesis. Scoring of d30 proximal colonic tissues for BrdU-positivity clearly showed that ablation of epithelial TGFβ-receptor signalling has no effect on epithelial cell proliferation (Fig. 4h). The elevated levels of apoptosis induced by mutant BRAF in vivo, however, were suppressed following epithelial-specific loss of TGFβ-receptor signalling, indicating reduced sensitivity to the initiation of cell death (Fig. 4i) as has previously been reported following loss of TGFβ-receptor signalling^49^. Taken together, these findings suggest that loss of TGFβ-receptor signalling allows colonic epithelial cells to evade apoptosis rather than augmenting their proliferative capacity.

### Right-sided tumorigenesis is supported by microbial-driven inflammation

**Fig. 5:**
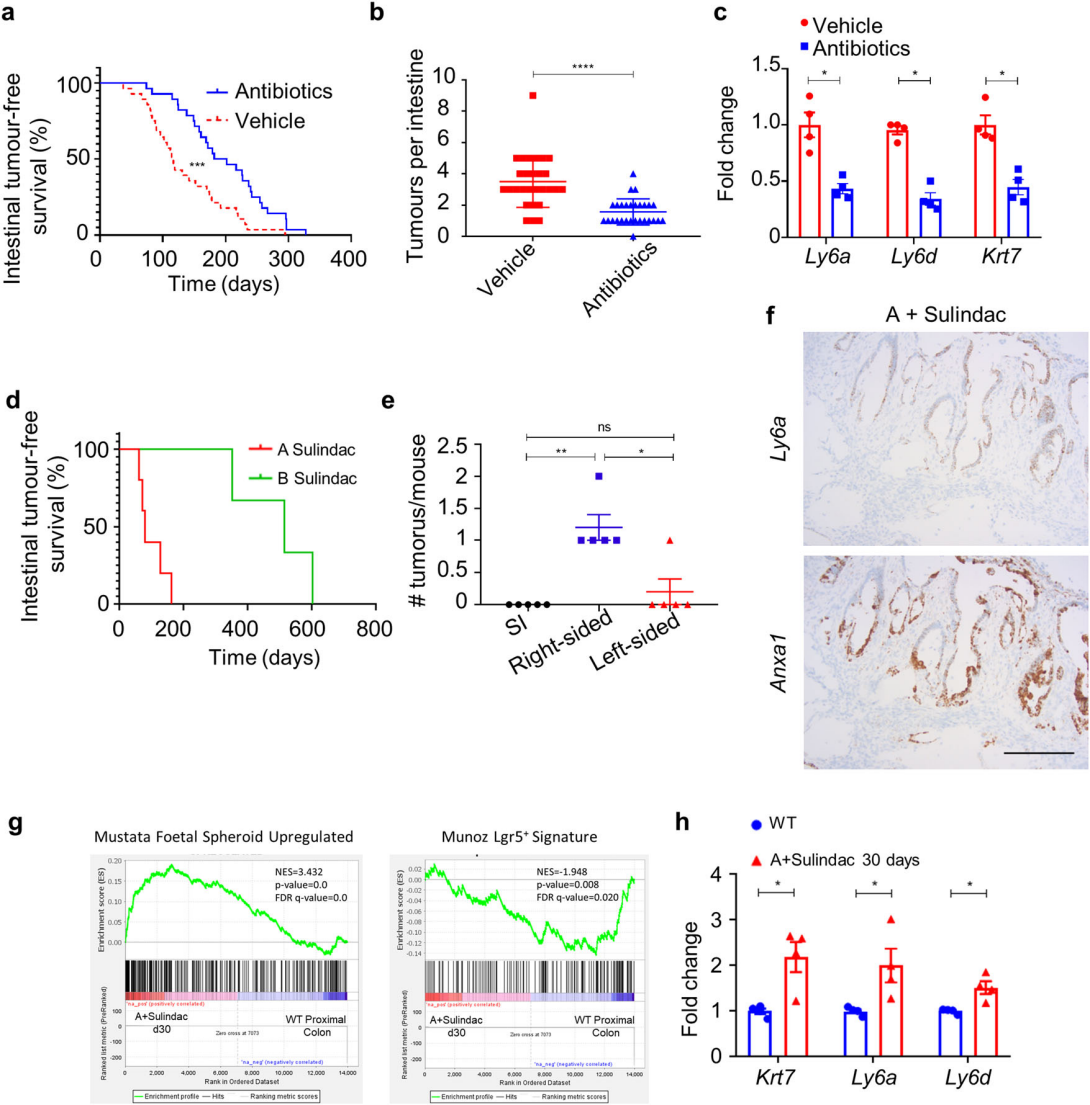
Inflammation and epithelial TGFβ-receptor loss cooperate in the formation of foetal-like tumours. **a** Kaplan–Meier survival curves highlighting tumour-free survival (days after tamoxifen-induction) of vehicle-treated (*n* = 28) and antibiotic-treated (*n* = 28) BA mice (***p = 0.0006; Mantel-Cox log-rank test, two-tailed). **b** Total number of intestinal tumours in vehicle-treated (*n* = 28) and antibiotic-treated (*n* = 28) BA mice. Mean ± s.e.m. (****p = 2.9e−7; Mann–Whitney *U*-test, two-tailed). **c** qRT-PCR of indicated foetal markers in proximal colonic tissue from vehicle- and antibiotic-treated BA mice, 30 days post induction (*n* = 4 mice per treatment). Fold change is shown relative to vehicle-treated BA mice Mean ± s.e.m. (*p = 0.0286; Mann–Whitney *U*-test, two-tailed). **d** Kaplan–Meier survival curves highlighting survival (days after induction) of sulindac-treated A (*n* = 5) and B (*n* = 3) mice. **e** Intestinal tumour location scoring of sulindac-treated A mice (*n* = 5). Mean ± s.e.m. (ns not significant; *p = 0.0397, **p = 0.0079; Mann–Whitney *U*-test, two-tailed). **f** Representative ISH of sulindac-treated A proximal colonic tumours, stained for foetal markers *Ly6a* and *Anxa1* (*n* = 5 mice). Scale bar, 100 μm. **g** GSEA plots of RNA-seq data from sulindac-treated A vs WT proximal colonic tissues, 30 days post induction, using the foetal spheroid (left panel) and *Lgr5*^+^ ISC (right panel) gene signatures (*n* = 4 mice per group). NES normalised enrichment score, FDR false discovery rate. (Left panel—*p* = 0.0, represents *p* < 0.001, Right panel—*p* = 0.008, calculated empirically.) **h** qRT-PCR of indicated foetal markers in proximal colonic tissue from sulindac-treated A mice vs WT counterparts, 30 days post induction (*n* = 4 mice per group). Fold change is shown relative to untreated WT mice. Mean ± s.e.m. (*p = 0.0286; Mann–Whitney *U*-test, two-tailed).

Our results indicate that mutant BRAF can initiate the foetal-like phenotype in organoids, via the activation of YAP, without additional genetic events or input from contextual cues. Nevertheless, the proximal colonic location of BA tumours—a region heavily colonized by intestinal microflora—and the presence of locoregional inflammation, evident from the early stages of tumour initiation, raised the possibility that local microenvironmental factors may influence tumorigenesis. Indeed, our data showed that the integrity of the mucosal barrier is compromised in BA tissues, likely allowing exposure of the epithelium to gut microbiota and toxic metabolites that can promote tumorigenesis by provoking inflammation^24,50^. To test this, we treated BA mice with broad-spectrum antibiotics from the time of tamoxifen-induction (Supplementary Fig. 6a). Antibiotics significantly extended survival and reduced the tumour number in BA mice, but this effect was only significant for right-sided tumours (Fig. 5a, b, Supplementary Fig. 6b). Comparison of RNA-seq analyses between antibiotic- and vehicle-treated d30 BA mice confirmed that eleven of the top fifteen pathways, significantly downregulated by antibiotic treatment, were associated with inflammation and the immune response (Supplementary Fig. 6c). Although still present, the foetal-like phenotype was dampened, with a reduction in the expression of foetal markers, including *Ly6a*, *Ly6d*, *Krt7*, and *Anxa1*, both in the tissue as a whole and in the epithelial compartment specifically (Fig. 5c, Supplementary Fig. 6d). These findings support the hypothesis that, although *BRAF* mutation alone drives foetal-like differentiation, microbial-driven inflammation is an additional key factor contributing to early tumour formation in the BA model, and that the local microbial milieu supports tumour initiation, right-sidedness, and disease progression. Our studies have, therefore, established a synergistic relationship between epithelial *Braf*^*V600E*^ mutation, loss of TGFβ-receptor signalling, and the local inflammatory microenvironment in driving right-sided, foetal-like tumours.

### Inflammation drives colonic tumorigenesis in a MAPK-independent manner

We next sought to model the effects of wounding and inflammation to determine whether these were sufficient to initiate and drive tumorigenesis in B or A mice. High-dose sulindac treatment has previously been shown to result in proximal colonic epithelial damage^51^. Therefore, to provide a brief window of inflammation/wounding, we administered sulindac for 14 days immediately post induction (Supplementary Fig. 7a). This acute insult was sufficient to cause tumour formation in the proximal colon of A but not B mice (Fig. 5d). Tumours in sulindac-treated A mice were almost exclusively right-sided (Fig. 5e), consistent with the location of the injury^51^. Similarly to the BA model, sulindac-treated A tumours were invasive, exhibited a mucinous histology, and stained strongly positive for foetal transcripts in the epithelial compartment (Fig. 5f, Supplementary Fig. 7b). In addition, proximal colonic tissue harvested from sulindac-treated A mice on d30 post induction (16 days after cessation of sulindac treatment) showed a significant positive enrichment for the foetal-like signature and a negative enrichment for the *Lgr5*^+^ ISC signature, as observed in BA mice (Fig. 5g, h, Supplementary Table 1).

Given the capacity of BRAF^V600E^-driven MAPK activation to engage a foetal-like program directly in vitro, we next addressed whether MAPK activation was responsible for the foetal-like differentiation emerging through sulindac-driven inflammation in vivo. Utilising the MEK-inhibitor AZD6244, we demonstrated that the instigation of the foetal-like program is likely not dependent on MAPK activation in this inflammatory context in WT mice (Supplementary Fig. 7c).

The ability of sulindac-induced inflammation to drive right-sided tumorigenesis in A mice mirrored the topography of emergent tumours in BA mice. Therefore, we next addressed whether we could influence the location of tumour formation by guiding inflammation to a different site. DSS has previously been shown to drive tumorigenesis in TGFβ-receptor knockout mice^52^. Using this model, we confirmed that DSS-induced inflammation elicited tumour formation in A mice. As expected, the emergent colonic tumours were invasive, displayed a mucinous histology, lacked Wnt-pathway activation, and expressed foetal transcripts in their epithelium. Importantly, these tumours were located more distally in the colon, as would be expected with DSS treatment (Supplementary Fig. 7d–f). Collectively, these findings suggest that, in the presence of inflammatory stimuli driving foetal-like epithelial differentiation, loss of TGFβ-receptor signalling is permissive for the emergence of invasive colonic tumours at the site of inflammation.

## Discussion

An improved understanding of the molecular pathogenesis of mutant BRAF-driven rCRCs will inform the development of effective preventative and therapeutic strategies for this aggressive CRC subset. Preclinical models that faithfully recapitulate the salient features of disease initiation and progression, as bestowed by different oncogenic drivers, are an important tool in this endeavour. Here, we describe a mouse model of serrated intestinal cancer wherein oncogenic BRAF and loss of epithelial TGFβ-receptor cooperatively promote the formation of right-sided colonic tumours with a Wnt-low, foetal-like signature. Emergent tumours in this model recapitulate the topography, molecular landscape, and metastatic tropism of human right-sided disease, lending important insights into the pathogenesis of mutant BRAF-driven rCRCs that arise via the serrated neoplasia pathway.

While mutant BRAF-driven serrated tumours show a clear predilection for the right side of the colon in humans, most existing mouse models develop lesions primarily in the small intestine rather than the colon^16–18^. Interestingly, Rad *et al*. found that BRAF^V600E^ induced widespread serrated hyperplasia throughout the intestinal tract. Yet, the vast majority of hyperplastic polyps that progressed to dysplasia in this model were located in the small intestine, with lesions exhibiting features of TSAs, rather than SSAs, which the authors attributed to the fact that SSAs typically emerge in the colon^16^. By contrast, BA tumours develop in the proximal/right colon and carry a Wnt-low, foetal-like signature which correlates with *BRAF*-mutant status, right-sidedness, MSI-high, CMS1 CRCs as well as pre-neoplastic SSAs in humans.

In further contrast to the BA model presented here, previous serrated tumorigenesis models exhibit deregulated Wnt signalling^16–18^. Notably, however, patient-derived right-sided serrated precursor lesions rarely harbour mutations in *APC* or *CTNNB1*, though mutations in lesser-known Wnt-pathway components can be acquired during the progression to dysplasia/more advanced stages^53–55^. Importantly though, BRAF^V600E^ right-sided colonic tumours in patients have been shown to display membranous not nuclear β-catenin localisation^19^, and we therefore suggest that our BA model better recapitulates this tumour group.

Previous studies employing an inducible human *BRAF*^*V600K*^ transgene resulted in epithelial serration and exhaustion/depletion of *Lgr5*^+^ ISCs, underpinned by their conversion to transit-amplifying (TA) progenitor cells^18^. By contrast, the depletion of *Lgr5*^+^ ISCs in the BA model is accompanied by the appearance of cells with a foetal-like phenotype. There are several differences between these models that may account for this discrepancy. First, the BA model develops SSA-like tumours in the proximal colon, whereas the transgenic *BRAF*^*V600K*^ model develops hyperplasia and TSA-like dysplastic polyps in the small intestine. Aside from region-specific differences in basal proliferation rates and crypt dynamics, the signals and niche cell-types that determine cell-lineage allocation—i.e., the interconversion between adult and foetal-like stem cell populations and TA-progenitor cell states/fates—vary profoundly lengthways along the intestinal tract and up the vertical crypt-axis^56^. Accordingly, BRAF^V600E^-mutant small intestinal and colonic organoids differ profoundly in basal MAPK-pathway activity, with small intestinal organoids able to tolerate higher pathway activity upon BRAF^V600E^ expression^57^. Notably, though, the transcriptomic profiles of BRAF^V600E^ colonic organoids more closely resemble human BRAF^V600E^-mutant CRCs^57^. Second, the BA model expresses physiological levels of a knock-in BRAF^V600E^ mutant driven by the endogenous *Braf* promoter, whereas the transgenic model relies on the overexpression of a human *BRAF*^*V600K*^ transgene, which attains expression of MAPK-target genes akin to levels in more advanced dysplastic tumours. A reduction in ISC markers was also found in the hyperplastic intestinal tissue of *BRAF*^*V637E*^ knock-in mice, which harbours comparable levels of MAPK activity to the transgenic model, although a TA cell fate was not expressly demonstrated^16,18^.

The conversion of the entire *Lgr5*^+^ ISC pool into TA progenitor cells, in the transgenic *BRAF*^*V600K*^ model^18^, is reminiscent of normal crypt homeostasis. Therein, reduced EGFR/MAPK activity at the crypt base prevents the quiescent ISC pool from differentiating prematurely, while elevated MAPK signalling in the upper crypt dictates a proliferative TA progenitor cell fate^58^. Although the determinants underlying the different fates of *BRAF*-mutant cells in these two models remain unclear, the implications are profound: While reversion of *Lgr5*^+^ ISCs to a foetal-like state, in the absence of TGFβ signalling, fuels aggressive BA tumour growth, conversion to a TA-like state leads to exhaustion of the cancer stem cell pool and clonal extinction. This likely explains the finding that, in the transgenic model, the progression of dysplastic foci was frequently accompanied by the acquisition of Wnt/β-catenin-pathway mutations, which restored ISC marker expression^18^. Indeed, this is consistent with studies showing that BRAF^V600E^ promotes loss of ISCs but, paradoxically, triggers differentiation; here, oncogenic progression is contingent on mutations that reverse differentiation (e.g. ablation of the differentiation-promoting transcription factors CDX2 or SMAD4) to restore stemness potential^59^. Accordingly, aging-induced acquisition of epigenetic alterations predisposes to BRAF^V600E^-driven transformation^60^, with several studies implicating cooperating inactivation of *Cdkn2a*/p16INK4a^16^, *Cdx2*^59,61^, or both atypical PKCs (PKCζ and PKCλ/ι)^62^ in BRAF^V600E^-induced tumorigenesis. Similarly, our studies identify epithelial TGFβ-receptor signalling as an important gatekeeper to BRAF^V600E^-driven right-sided tumorigenesis. Indeed, we also show that the tumorigenic potential of foetal-like populations in the right colon is suppressed by epithelial TGFβ-receptor signalling, independent of the factors driving the foetal-like differentiation program, be it an acute inflammatory insult or aberrant MAPK activation. Indeed, this strongly suggests that the co-occurrence of *TGFBR2* and *BRAF* mutations in patient rCRCs^30^ provides foetal-like right-sided epithelial cells with a selective advantage.

Our study further shows that the MAPK pathway, via ERK, is fundamentally capable of driving a foetal-like differentiation program in the colonic epithelium in a YAP-dependent manner, and that this is further augmented in vivo by microbial-driven inflammation. Controversy surrounds the role of YAP in intestinal tumorigenesis, with both tumour-suppressive^63,64^ and oncogenic roles^23,65–67^ ascribed in different contexts. Several studies have demonstrated that YAP can inhibit Wnt signalling during intestinal regeneration and tumorigenesis^23,63,64,68^, consistent with the suppression of *Lgr5* and the ISC signature in our BA model. In strong support of our findings, while this paper was under review, Reischmann *et al*. reported that the expression of BRAF^V600E^ in colonic organoids elicits a foetal-like dedifferentiation program, associated with a transcriptional signature enriched for Hippo-pathway targets that resembles human BRAF^V600E^-driven CRC^57^. Interestingly, activation of the ERK and YAP pathways has also been detected in an inflammation-driven model of serrated intestinal tumorigenesis arising through atypical-PKC deficiency, independently of *Braf* (and *Kras*) mutations^62^, signifying that YAP dysregulation may commonly underlie distinct serrated neoplasia pathways independently of the driver mutation.

As multiple *Lgr5*^+^ and *Lgr5*^−^ populations exhibit phenotypic plasticity and tumour-initiating potential within the intestine, questions remain as to the likely cell(s)-of-origin of the emergent tumours in the BA model and the serrated neoplasia pathway. A recent study identified a rare, quiescent population of revival stem cells, distinguished by elevated expression of clusterin (*Clu*) as well as *Anxa1*, *Cxadr*, and *Basp1*, that is mobilized and expanded following damage to the intestinal epithelium, regenerating all the major intestinal cell types, including *Lgr5*^+^ ISCs, in a YAP1-dependent manner^69^. It remains unclear whether different crypt progenitors and/or mature cell types are able to dedifferentiate to a foetal-like state in response to BRAF activation, or whether homeostatic colonic crypt subpopulations, such as the revival stem cells, acquire *Braf* mutation and initiate serrated tumorigenesis independently of Wnt signalling: the answer awaits lineage-tracing and single-cell genomics approaches in our BA model.

Our data have allowed us to identify and highlight the diverse nature of left and right-sided CRC in patients, showing that they are underpinned throughout tumour initiation and progression by different signalling pathways, which lead to either a Wnt high, Lgr5^+^ tumour phenotype or a BRAF-mutant, foetal-like, Lgr5^-^/Wnt-low phenotype, respectively. rCRCs that harbour oncogenic BRAF, however, continue to pose a major therapeutic challenge, with patients deriving little to no benefit from prevailing treatment regimens. While BRAF inhibitors have shown considerable efficacy in melanoma^70^, BRAF-mutant rCRCs are refractory to such treatment owing to EGFR-driven feedback activation of the MAPK pathway^71,72^. Of note, the YAP-driven regenerative response in the injured intestinal epithelium promotes cell survival by activating EGF signalling^23^. Accordingly, elevated YAP expression is predictive of intrinsic and acquired resistance to BRAF- and MEK-inhibitors in human BRAF^V600E^ CRCs^73^, lending clinical relevance to our finding that YAP is a survival factor in BA tumours, and advocating for concurrent inhibition of YAP and BRAF-MAPK signalling in this setting. Moreover, a recent study in compound mutant organoids, with *Rspo3*-fusions and *Kras*/*Braf*, *Trp53*, and *Smad4* mutations, implicated YAP/TAZ foetal reprogramming and lineage reversion in the acquisition of resistance to Wnt-targeted therapy. Significantly, emergent organoids were addicted to YAP/TAZ signalling and were sensitive to YAP/TAZ inhibition^74^. Encouragingly, foetal-like colonic BA tumour epithelium also maintains a dependence on therapeutically targetable, microenvironmental-derived inflammatory factors, such as PGE2. Additionally, the absolute requirement for YAP activation in the intestinal epithelium after *BRAF* mutation provides a further novel potential therapeutic target downstream of BRAF activation.

Our BA model recapitulates the topography, molecular landscape, and metastatic tropism of human Wnt-low, *BRAF*-mutant rCRCs and offers potential actionable therapeutic targets for this aggressive therapy-resistant subtype.

## Methods

### Mouse studies

All animal experiments were performed in accordance with UK Home Office regulations (Project licence 70/8646), with adherence to the ARRIVE guidelines, and were reviewed and approved by the Animal Welfare and Ethical Review Board (AWERB) of the University of Glasgow. Mice used were of a C57BL/6 J background (*N* ≥ 6) and harboured combinations of the following genotypes: the VillinCre^ER^ transgene^26^ *Apc*^*fl/+*^^33^, *Braf*^*LSL-V600E/+*^ (hereafter *Braf*^*V600E/+*^)^27^, *Tgfbr1/Alk5*^*fl/fl*^^28^, *Tgfbr2*^*fl/fl*^^31^, *Yap1*^*fl/fl*^^75^, and *Rosa26*^*LSL-tdTomato/+*^^76^. For the generation of *Mlh1*^*fl/fl*^ mice, embryos carrying the *Mlh1*^*tm1a*^ allele were imported from the European Mouse Mutant Archive (EM:04517). To generate the floxed allele, mice derived from these embryos were subsequently crossed with a strain expressing Flpe (B6.Cg-Tg(ACTFLPe)9205Dym/J; JAX stock #005703) to delete the lacZ reporter and neomycin resistance (lacZ/neo^R^) cassette by recombination at the flanking FRT sites^77^. Correct removal of the lacZ/neo^R^ cassette was confirmed by PCR across the remaining FRT site (Fwd, 5′-GTAATACTCTCTAGAGGCCCATGC-3′ and Rev, 5′-TATTAGCTCGTGGTTTTAGATGACC-3′; amplicon: 404 bp). Following Flpe-mediated recombination, critical exon 4 of *Mlh1* is flanked by loxP sites, generating a floxed *Mlh1*^*tm1c(EUCOMM)Hmgu*^ allele that can be conditionally deleted in the intestine upon crossing with VillinCre^ER^ mice.

Mice were induced between 6 and 12 weeks of age and sampled at the indicated timepoints or at clinical endpoint. Unless otherwise stated, mice were induced by either a single intraperitoneal injection of 2 mg tamoxifen (80 mg/kg; Sigma, T5648), or two injections of 3 mg (120 mg/kg) and 2 mg (80 mg/kg) tamoxifen on consecutive days. Mice were monitored daily and weighed a minimum of three times weekly and humanely culled upon reaching clinical endpoint, defined by symptoms associated with intestinal tumour burden. Mice were censored at 550 days after initial tamoxifen-injection.

### In vivo drug treatments

For antibiotic treatment, the antibiotic combination used consisted of ampicillin (1 g/L; Sigma, A9518), neomycin trisulphate (1 g/L; Sigma, N6386), vancomycin (500 mg/L; Wockhardt, 17116), and metronidazole (1 g/L; Sigma, M1547), dissolved in distilled water. From the first day after the initial tamoxifen-injection, mice were treated with antibiotics in their drinking water, which was changed three times weekly. High-dose sulindac was administered in the drinking water (0.4 g/L in sodium phosphate buffer; Sigma, S8139) from the first day after the initial tamoxifen-injection, for a total of 14 days, before returning the mice to normal drinking water. Sulindac, in drinking water, was delivered in dark bottles, which were changed weekly along with the sulindac. For the induction of inflammation, mice received dextran sulphate sodium salt (DSS; MP Biomedicals, 160110) in distilled drinking water on day 3 after the initial tamoxifen-injection. DSS (1.5% w/v in drinking water) was administered *ad libitum* for five days and, subsequently, withdrawn and replaced with fresh distilled water. For in vivo FAK inhibition^40,41^, mice were treated with 18.75 mg/kg VS-4718 reconstituted in 0.5% hydroxypropyl methylcellulose (HPMC) + 0.1% Tween-80 vehicle by oral gavage once daily. For in vivo MEK inhibition^43,44^, mice were treated with 25 mg/kg AZD6244 made up in the same vehicle as VS-4718, but administered by twice-daily oral gavage.

### FITC-dextran gut permeability assay

Mice were induced with two injections of 3 mg (120 mg/kg) and 2 mg (80 mg/kg) tamoxifen on consecutive days. On day 7 post induction, mice were fasted for 12 h and then dosed with 600 mg/kg FITC-dextran (4 kDa; Sigma, #46944) by oral gavage. Mice were sacrificed and blood was sampled 4 h post gavage. Serum concentrations of FITC-dextran were determined using a fluorimeter (490/530 nm) against standard curves generated from the same stock solution used to dose the mice.

### Immunohistochemistry, in situ hybridization, and histopathology

Intestinal tissues were fixed in 10% neutral-buffered formalin overnight and embedded in paraffin. Haematoxylin-and-eosin (H&E) staining was performed using standard protocols. Immunohistochemistry (IHC) for cytokeratin-7 (1:100; Abcam, ab9021), cleaved caspase 3 (1:500; Cell Signaling Technology, 9661), BrdU (1:150; BD Biosciences, 347580), and RFP (1:100; Cell Signaling Technology, 2555), to detect tdTomato and evaluate the efficiency of Cre-mediated recombination, were performed on 3 μm sections with heat-based antigen retrieval at pH 6 and pH 8, respectively. For β-catenin IHC, fixation was performed at 4 °C for no more than 24 hours, and sections were immunostained with antibodies against β-catenin (1:50; BD Biosciences, 610154)^78^. RNA in situ hybridisation (ISH) was performed using the RNAscope 2.5 LS Reagent Kit–BROWN (Advanced Cell Diagnostics, 322100) on a Leica Biosystems BOND RX automated IHC/ISH stainer according to the manufacturer’s instructions. Positive control probes (Mm-PPIB; Advanced Cell Diagnostics, 313918) were run to ensure RNA integrity in analyzed samples. Negative control probes were run to ensure the specificity of the staining (dapB; Advanced Cell Diagnostics, 312038). The RNAscope probes used were: *Notum* (428988), *Axin2* (400338), *Lgr5* (312178), *Ly6a* (427578), and *Anxa1* (509298).

Tumours were scored by gross examination of the excised tissue and histopathological analysis of H&E-stained sections. The proximal/right side of the colon was defined as the region extending from the ileocolic junction to the distal end of the region of the colon containing prominent transverse folds. The left side consisted of all colonic regions distal to this point. H&E-stained intestinal sections were evaluated for sub-cryptal proprial neutrophil infiltration by a boarded pathologist (J.D.G.L.). Neutrophils were identified by nuclear morphology and cytoplasmic tinctorial properties. The numbers of neutrophils within the band of lamina propria, immediately beneath and surrounding the colonic crypts, were quantified by image capture and analysis of H&E-stained tissue sections. Normalizing to the width of a 400x magnification field of view (FOV) of the colon crypts and the surrounding lamina propria allowed for the quantification and normalisation of neutrophil numbers within an area of tissue likely to expand in depth following significant immune infiltration. Ten to twenty 400x FOV were scored per region depending on length of region.

Using RFP immunohistochemistry, the efficiency of Cre-mediated recombination was scored in VillinCre^ER^; *Rosa26*^*LSL-tdTomato/+*^ mice receiving escalating tamoxifen-dosing regimens, as indicated in Supplementary Fig. 1c. Crypts were scored as positive when an area greater than, or equal to, half of the crypt stained positive for RFP. At least 50 crypts were scored per mouse per tamoxifen-dosing regimen in 3 independent VillinCre^ER^; *Rosa26*^*LSL-tdTomato/+*^ mice.

Immunohistochemistry images were scanned and subsequently analyzed using HALO software (V2.0.1145, Indica Labs). Proximal colonic epithelium was manually annotated to include all epithelium visible and then analyzed for the percentage of positive cells for cleaved caspase 3 or BrdU.

### RNA isolation from tissue

Tumours and proximal colonic tissues were harvested into RNA*later* and snap-frozen on dry ice. Tissues were homogenized using the Precellys lysing kit (Bertin Instruments, KT03961-1-003-2) in a Precellys Evolution tissue homogenizer. RNA was extracted and purified, using the Qiagen RNeasy Mini kit (Qiagen, 74104), as per the manufacturer’s protocol, including all optional steps.

### qRT-PCR

cDNA was generated using the High-Capacity cDNA Reverse Transcription Kit (ThermoFisher Scientific, 4368814) as per the manufacturer’s protocol. qPCR was performed using the Maxima SYBR Green/ROX qPCR Master Mix (ThermoFisher Scientific, K0221) as per the manufacturer’s protocol on Applied Biosystems StepOnePlus PCR system using StepOne software version 2.3 (qPCR). Ct-values were normalised to *Gapdh* transcript levels. mRNA expression levels were calculated via the ΔΔCt method utilising Microsoft Excel 2016. For primer sequences please see Supplementary Table 2.

### RNA-seq and data analysis

RNA quality was ensured utilising the Agilent 2200 TapeStation System and RNA ScreenTape (Agilent, 5067-5576). Libraries for DNA sequencing were prepared utilising a method adapted for the Illumina TruSeq RNA LT Kit. The quality of DNA libraries was established using the Agilent 2200 TapeStation. Library sequencing was run on the Illumina NextSeq 500 desktop sequencer using the High Output 75-cycle kit (2 ×36 cycles). Quality checks on the raw RNA-Seq data files were done using FastQC version 0.11.8, then sequences were trimmed to remove adaptor sequences and low-quality base calls, defined as those with a Phred score of less than 20, using Trim Galore version 0.6.4. Trimmed paired-end reads were then aligned to the GRCm38.98 version of the *Mus musculus* genome using HISAT2 version 2.1.0 and raw counts per gene were determined using FeatureCounts version 1.6.4. Differential expression analysis, based on the negative binomial distribution, was performed using the R package DESeq2 version 1.22.2. Gene set enrichment analyses (GSEA) were performed on pre-ranked RNA-seq datasets using the Broad Institute GSEA tool (software.broadinstitute.org/gsea/index.jsp) with standard settings. The gene sets used included the foetal spheroid upregulated signature^21^, the Hallmark Inflammatory_Response gene set (Broad Institute)^37^, the YAP up- and down-regulated signatures^23^, and the *Lgr5* intestinal stem cell signature^22^. Pathway analysis was performed utilising MetaCore (Clarivate Analytics).

### Organoid cultures

Advanced DMEM/F12 was supplemented with penicillin (100 U/ml) and streptomycin (100 µg/ml) (ThermoFisher Scientific, 15140122), 2 mM L-glutamine (ThermoFisher Scientific, 25030081), 10 mM HEPES (ThermoFisher Scientific, 15630080), N2-supplement (ThermoFisher Scientific, 17502001) and B27-supplement (ThermoFisher Scientific, 17504044) and designated as ADF. Complete ADF was prepared from ADF by adding 50 ng/ml recombinant human EGF (Peprotech, AF-100-15), 100 ng/ml recombinant murine Noggin (Peprotech, 250-38), and 500 ng/ml recombinant mouse R-spondin-1 (R&D systems, 3474-RS). For wild-type colonic organoid culture, complete ADF was further supplemented with 2.5 μM CHIR99021 (Sigma, SML1046), 1 μM valproic acid (Sigma, P4543), and 10 μM Y-27632 (Cambridge Bioscience, SMO2-1). For serial passaging of induced (tamoxifen-treated) or tumour BA organoids, the medium was further supplemented with 2.5 μM PGE2 (Tocris Bioscience, 2296).

For in vitro BA organoid drug treatments, organoids were induced with 5 μM 4-hydroxytamoxifen (Sigma, #T5648) 24 h post passage. BA organoids were then treated with drug 48 h post induction and harvested for analysis 48 h post drug addition. All drugs were made up in DMSO, at a final concentration of 0.1%, which served as a vehicle control. Final drug concentrations were: VS-4718^40,41^ (FAKi) at 1 μM, eCF506^42^ (SRCi) at 50 nM, AZD6244^43,44^ (MEKi) at 100 nM, ERKi^45^ at 100 nM, and verteporfin^20^ (YAPi) at 3 μM.

Colonic organoid isolation and culture were performed as previously reported^79^. Briefly, for the isolation of tumour organoids, murine intestinal tumour tissue was sampled at a defined endpoint, cut into small fragments with forceps and scissors, and washed five times in PBS. Tumour fragments were incubated in 5 ml of 10x trypsin (5 mg/ml; Gibco), 1x DNase I buffer, and 200 U recombinant DNase I (Roche, 04716728001) for 30 minutes in a 37 °C water bath. Tumour fragments were, then, further dissociated in 5 ml ADF with vigorous shaking. This step was repeated five times. The tissue suspension was then centrifuged at 260 *g*, and the supernatant was aspirated and the pellet was resuspended in 10 ml ADF. The resulting suspension was then passed through a 70 µm cell strainer and centrifuged at 260 × *g*. The cell pellet was resuspended in an amount of Matrigel (BD Bioscience, 354234), proportional to the pellet volume, and seeded. Organoids/spheroids were cultured in complete ADF supplemented with a cocktail of growth factors appropriate to each genotype, as detailed above, and maintained at 37 °C in a humidified atmosphere of 5% CO_2_ and 21% O_2_.

### Organoid immunofluorescence

Organoid immunofluorescence was performed in accordance with previously published methods^80^. Briefly, whole organoids were dissociated from matrigel, washed in PBS and fixed with 4% PFA on ice. Organoids were blocked for 1 hr at room temperature (1% BSA/PBS) and stained overnight at 4 °C with the following primary antibodies: rat anti-SCA1/LY6A (1:100; R&D Biosystems, 177228) and rabbit anti-YAP (1:100; Cell Signaling Technology, 4912). Organoids were exposed to Alexa Fluor 488 Goat anti-rabbit IgG or Alexa Fluor 594 goat anti-rat IgG (Invitrogen, 1/500) 2 hours at room temperature and counterstained with DAPI. Z-stack fluorescent and brightfield images were taken on a Zeiss 710 confocal microscope. Image analysis and Z-projection were performed using FIJI software and brightness and contrast settings were maintained between control and test images.

### Statistical analyses

Statistical analyses were performed using GraphPad Prism software (v8.02; GraphPad Software Inc.) and R (version 3.6.0; www.r-project.org/). Generation of gene signatures through differential gene expression analysis was performed with edgeR version 3.28.1 and the limma R package^81^. Human orthologues of mouse genes were obtained from Ensembl in March 2020. T-test and Mann–Whitney *U*-tests were used to determine statistical significance as appropriate. Survival analysis was based on Kaplan–Meier curves and univariable Cox-models were built for statistical testing with the log-rank test.

### CRC-patient data for in silico analysis

CRC-patient data were obtained from a range of publicly available sources. Expression data and clinical/genetic annotation from the TCGA project^82^ were downloaded from the FIREHOSE repository (https://gdac.broadinstitute.org/). Patient-derived CMS signatures were derived from the TCGA dataset (https://www.cancer.gov/tcga). Gene expression data from human colorectal tumours, sampled during the PETACC-3 trial, were found in ArrayExpress (Accession No: E-MTAB-990)^34^. Single-cell RNA-seq data, used to derive a list of epithelial-specific genes, can be found in ref. ^35^. The polyp dataset (*n* = 44) was collected by the S:CORT consortium (Ethics: MREC 15/EE/0241). Data relating to transcriptome profiling of relevant genes are available online (https://www.s-cort.org/)^39^.

### BA and WA signature derivation

The derivation of gene expression signatures was based on genes that were differentially expressed in mouse datasets at an absolute logFC>2 and an unadjusted *p* value of <0.00001 in an edgeR model incorporating batch and gene activation status. These genes were mapped to the most confident human orthologues, based on the Ensembl annotation (March 2020). The genes were further classified into epithelial, non-epithelial, and non-specific, according to the specificity of their expression in a previously published human colorectal single-cell dataset^35^. Briefly, cells were annotated into major types with SingleR. Then, raw counts from cells of a similar type (epithelial, stroma, myeloid, lymphocytes, endothelial, and other) were aggregated into pseudobulk with the muscat package. If the expression of a gene was higher in a given cell type than in all other cell types by at least 1 raw count, and that difference was always statistically significant at *p* < 0.01, the gene was considered specific for that cell type.

For the BA signature, both an upregulated and downregulated epithelial component were retained, and the difference in their mean expression levels comprised the signature value. For the WA signature, in order to increase specificity, the upregulated epithelial component was mapped to a heatmap of human colorectal cancer bulk gene expression data (E-MTAB-990 in ArrayExpress) with hierarchical clustering on correlation distance and complete linkage. The most significant cluster of epithelial genes was centred on *AXIN2* in the human data and was considered specific for Wnt activation. The mean expression of the genes (*MACC1, KRT23, FGFR4, RPS6KA6, PROX1, ASCL2, AXIN2, RASSF10*) constitutes the WA signature.

### Reporting summary

Further information on research design is available in the Nature Research Reporting Summary linked to this article.

## Supplementary information

Supplementary Information

Reporting Summary

## Data Availability

All data relevant to this study are available from the authors at reasonable request. The RNA-seq datasets that support the findings described here have been deposited at NCBI Gene Expression Omnibus (https://www.ncbi.nlm.nih.gov/geo) with the study accession code GSE168478. All source data are available as a Source Data file. The remaining data are available within the Article, Supplementary Information or from the authors upon request. Source data are provided with this paper.

## Source data

Source Data
