# Supplementary material for: Oncogenic BRAF, unrestrained by TGFβ-receptor signalling, drives right-sided colonic tumorigenesis
Source: Nat Commun. 2021 Jun 8;12:3464. doi: 10.1038/s41467-021-23717-5 (PMC8187652; doi:10.1038/s41467-021-23717-5)
Supplement: Supplementary file 1 — Supplementary Information [file 41467_2021_23717_MOESM1_ESM.pdf]

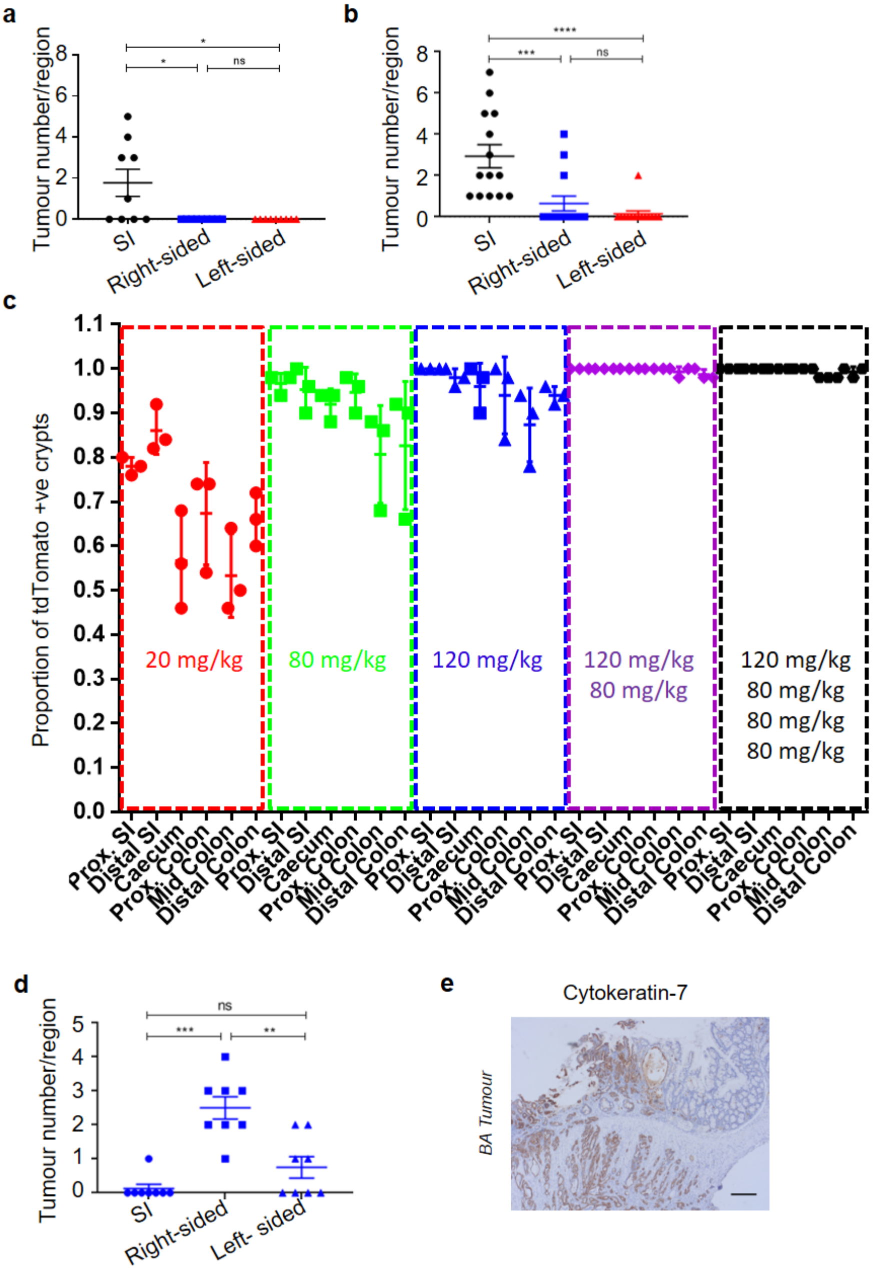

Supplementary Fig. 1: *$Braf^{V600E}$* and epithelial TGFβ-receptor loss cooperatively promote right-sided tumorigenesis

a, Intestinal tumour location scoring of VillinCre^{ER}; *$Braf^{LSL-V600E/+}$* (B) mice (n=9). Mean ± s.e.m. (ns, not significant; **p*=0.0294; Mann–Whitney *U*-test, two-tailed). **b**, Intestinal tumour location scoring of VillinCre^{ER}; *$Mlh^{fl/fl}$* mice (n=14). Mean ± s.e.m. (ns, not significant; ****p*=0.0002, *****p*=<0.0001; Mann–Whitney *U*-test, two-tailed). **c**, Cre-mediated recombination efficiency in VillinCre^{ER}; *$Rosa26^{LSL-tdTomato/+}$* crypts along the length of the small intestine, caecum, and colon. Recombination efficiency was scored for 50 crypts per mouse at each intestinal location in each of 3 mice at the indicated tamoxifen-induction dose/regimen (mg/kg per day; administered once every 24 h for one to four consecutive days, as indicated). n=3 mice per dosing regimen. Mean ± s.e.m. **d**, Intestinal tumour location scoring of VillinCre^{ER}; *$Braf^{V600E/+}$*; *$Tgfbr2^{fl/fl}$* mice (n=8). Mean ± s.e.m. (ns, not significant; ***p*=0.0059, ****p*=0.0003; Mann–Whitney *U*-test, two-tailed). **e**, Representative immunohistochemical staining of BA intestinal tumour epithelium for the foetal marker cytokeratin-7 (n=5 mice). Scale bar, 500 μm.

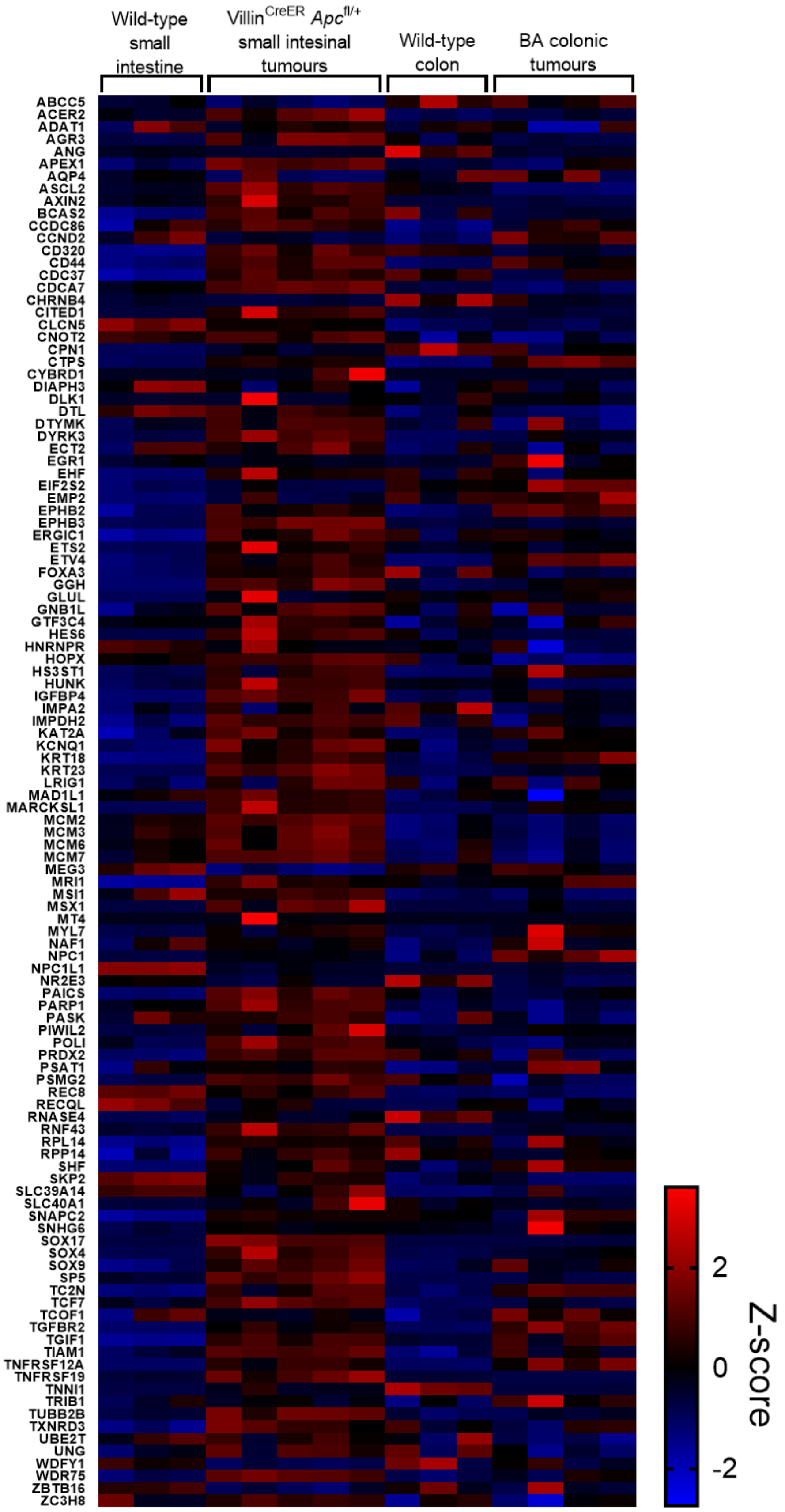

Supplementary Fig. 2: Genes upregulated following Wnt activation are enriched in *Apc* deficient tumours and not in BA tumours.

Heatmap of Z-score-transformed relative expression levels of genes upregulated upon homozygous deletion of *Apc* in the murine intestinal epithelium, in either BA proximal colonic tumours (n=4) vs WT proximal colon (n=3), or $Apc^{fl/+}$ small intestinal tumours (n=5) vs WT small intestine (n=3).

a

WA signature – genes upregulated in epithelium
MACC1
KRT23
FGFR4
RPS6KA6
PROX1
ASCL2
AXIN2
RASSF10

b

BA signature - genes up regulated in epithelium		BA signature - genes downregulated in epithelium	
PRSS22	RASAL1	UGT8	PAPSS2
TRIM29	LPCAT4	PBLD	ASPG
SLC4A11	MARCKSL1	NMU	SLC16A9
CEMIP	RANBP17	SULT1C2	SYBU
S100A14	PRRG4	BEST2	HKDC1
MFSD2A	PHLDA2	NAGS	
PDX1	VILL	SELENBP1	
ETV4	PCSK9	CYP2W1	
PGAP1	MACC1	CLDN8	
RNF183	SAMD5	SH3RF2	
ANXA3	S100A6	ARG2	
LAMA3	KRT7	HMGCS2	
LAMC2	S100A16	PRLR	
SCEL	PLEK2	MNX1	
EPHA2	ITGB4	FGFBP1	
GJB5	BTC	ITPKA	
CYSRT1	PLCD3	VSIG2	

c

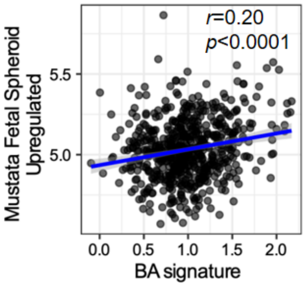

d

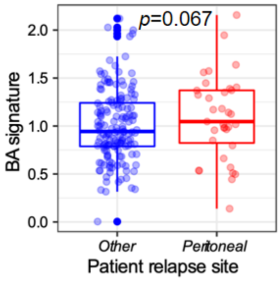

Supplementary Fig. 3: Gene signatures of murine right-sided BA tumours, at early timepoints, are predictive of rCRC clinical behaviour.

a. Wnt activation (WA) gene signature generated from datasets representing deletion of *Apc* in the murine intestinal epithelium. **b.** BA gene signatures representing genes up- or downregulated in BA endpoint tumour and BA d30 timepoint samples. **c**, Higher values of the BA signature in human tumours are weakly but significantly associated with the expression of the Mustata *et al*. foetal spheroid upregulated genes (Pearson's r=0.2 p=5.04e-7) (n=461). The regression line is presented with 95% confidence intervals (shaded area). **d**, In patients who relapsed, BA signature values tended to be higher when the relapse was peritoneal, compared with all other relapse sites, although the difference is not significant (p=0.067; T-test, two-tailed). (n=190). Median is shown with boxes, which extend from the 25th to the 75th percentile and whiskers which extend an additional 1.5x the interquartile range. Any value beyond these ranges are shown as outliers.

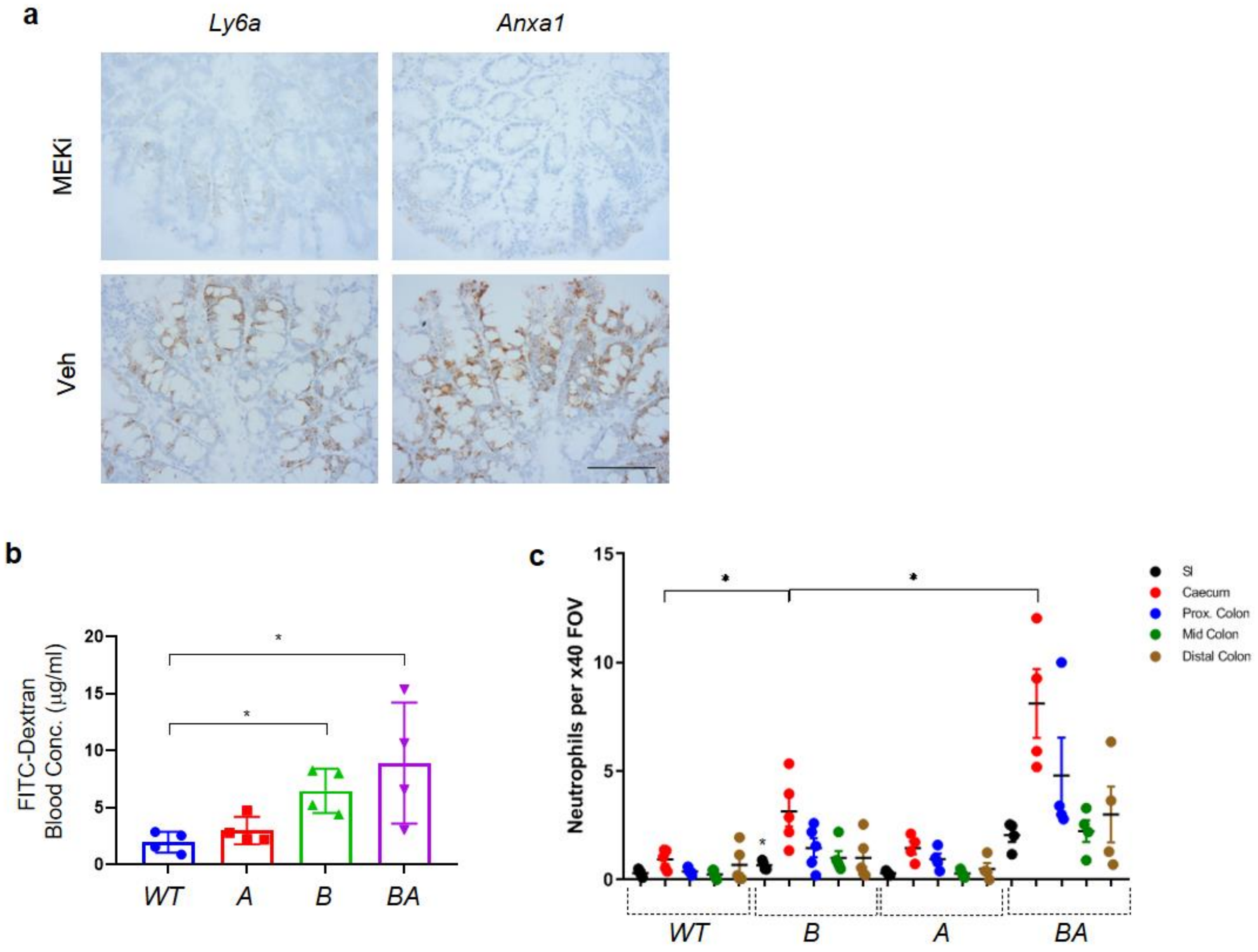

Supplementary Fig. 4: *Braf*^{V600E} elicits MEK-dependent foetal marker expression increasing intestinal permeability and regional inflammation.

a, Representative ISH of MEKi- (AZD6244) and vehicle-treated BA proximal colonic tissues, 30 days post induction, stained for foetal markers *Ly6a* and *Anxa1* (n=5 mice). Scale bar, 100 µm. **b**, Intestinal permeability measured by quantification of serum FITC-dextran in WT, A, B, and BA mice, 7 days post induction (n=4 mice per group). Mean ± s.e.m. (*p=0.0194; Mann–Whitney U-test, two-tailed). **c**, Location-specific infiltration of neutrophils into the intestinal lamina propria of the indicated intestinal regions in WT, B, A, and BA mice, 30 days post induction (n=4 mice per group). Mean ± s.e.m. (*p=0.0194; Mann–Whitney U-test, two-tailed). FOV, field of view.

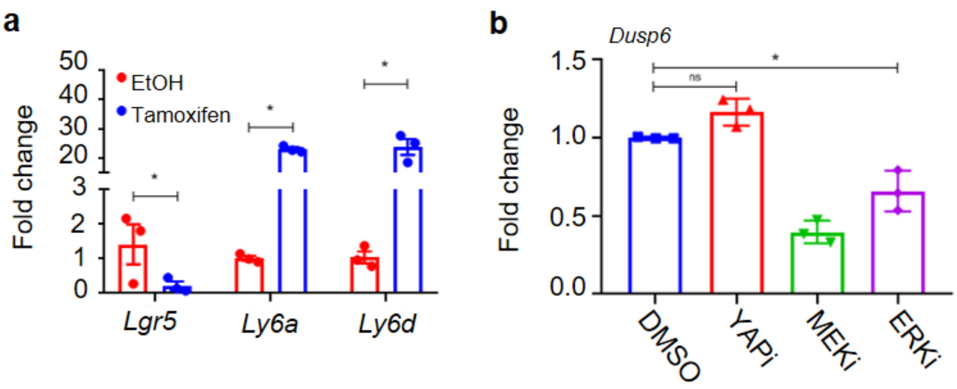

Supplementary Fig. 5: *Braf^{V600E}* mutation leads to upregulation of foetal marker expression *in vitro*.

a, qRT-PCR of *Lgr5* and indicated foetal markers in B organoids, induced *in vitro* with 4-hydroxytamoxifen and sampled 5 days post induction. n=3 organoid lines from 3 independent uninduced B mice. Fold change is shown relative to uninduced (EtOH-treated) organoids. Mean ± s.e.m. (**p*=0.05; Mann–Whitney *U*-test, two-tailed). **b**, qRT-PCR of *Dusp6* expression in BA organoids induced *in vitro* with 4-hydroxytamoxifen and sampled 48 h post drug-treatment, 5 days post induction. Drugs were reconstituted in DMSO vehicle (0.1%) at the indicated final concentrations: verteporfin (YAPi; 3 µM), AZD6244 (MEKi; 100 nM), and ERKi (100 nM). n=3 organoid lines from 3 separate uninduced BA mice. Fold change is shown relative to induced DMSO-treated organoids. Mean ± s.e.m. (ns, not significant; **p*=0.05; Mann–Whitney *U*-test, two-tailed).

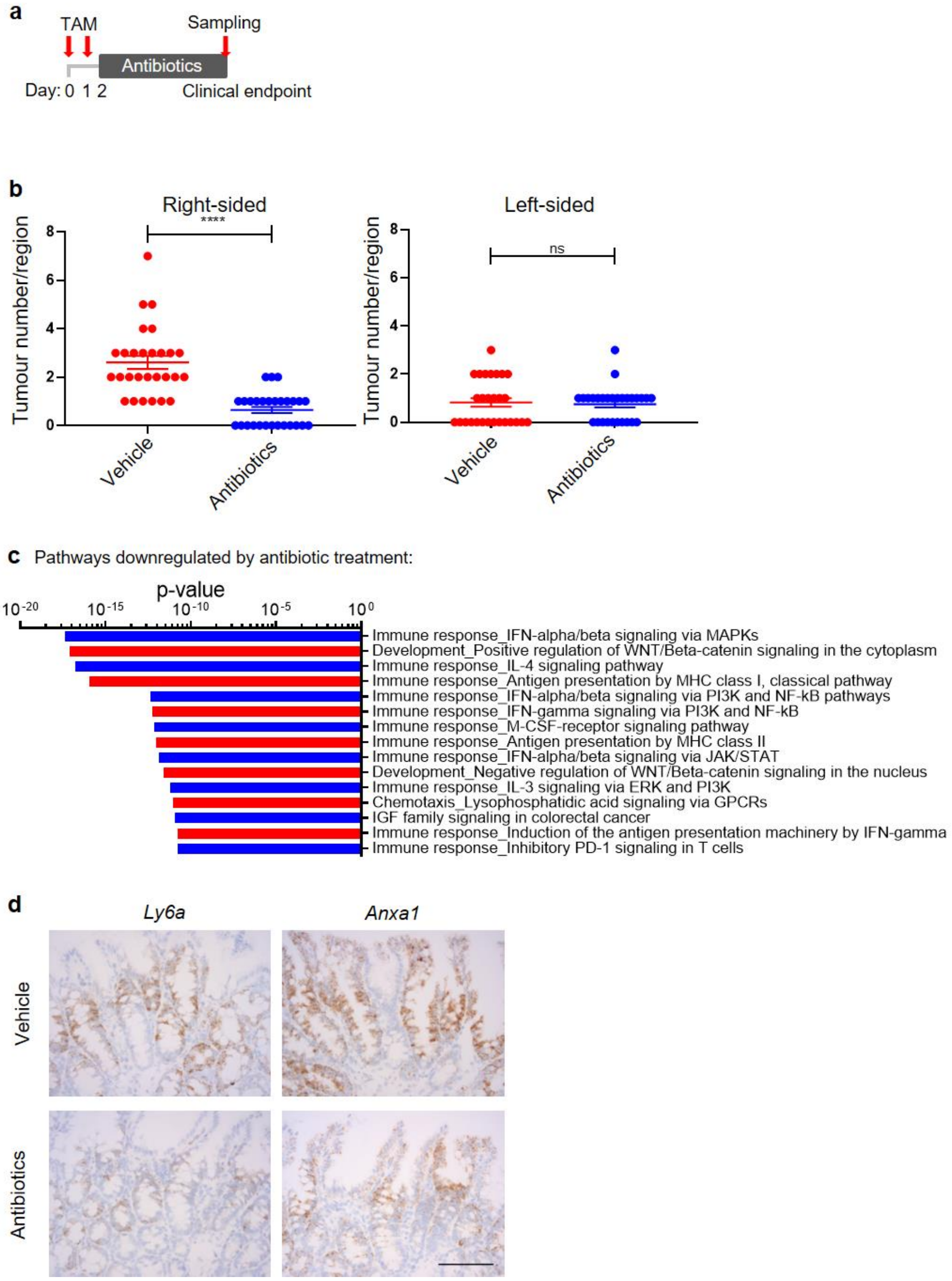

Supplementary Fig. 6: Microbiome-driven inflammation promotes foetal marker expression and tumorigenesis in the proximal colon.

a, Schematic outlining tamoxifen (TAM) induction and broad-spectrum antibiotic treatment of BA mice. An initial dose of 3 mg TAM (120 mg/kg) was administered on day 0, followed by 2 mg (80 mg/kg) on day 1. Daily antibiotic treatment was initiated from day 2 and continued until clinical endpoint. **b**, Quantification of colonic tumours by location in vehicle-treated (n=28) and antibiotic-treated (n=28) BA mice. Mean ± s.e.m. (ns, not significant; ****p=1.6e-9; Mann–Whitney U test, two-tailed). **c**, MetaCore pathway analysis indicating the most significantly downregulated pathways in proximal colonic tissue in antibiotic- vs vehicle-treated BA mice, 30 days post induction (n=4 mice per treatment). The downregulated pathways are indicated on the right side of the graph and are ranked by p-value, with the bar length indicating the level of significance; p-values generated from hypergeometric distribution test. **d**, Representative ISH of proximal colonic tissue from vehicle- and antibiotic-treated BA mice, 30 days post induction, stained for foetal markers *Ly6a* and *Anxa1*. n=5 mice per treatment. Scale bar, 100 µm.

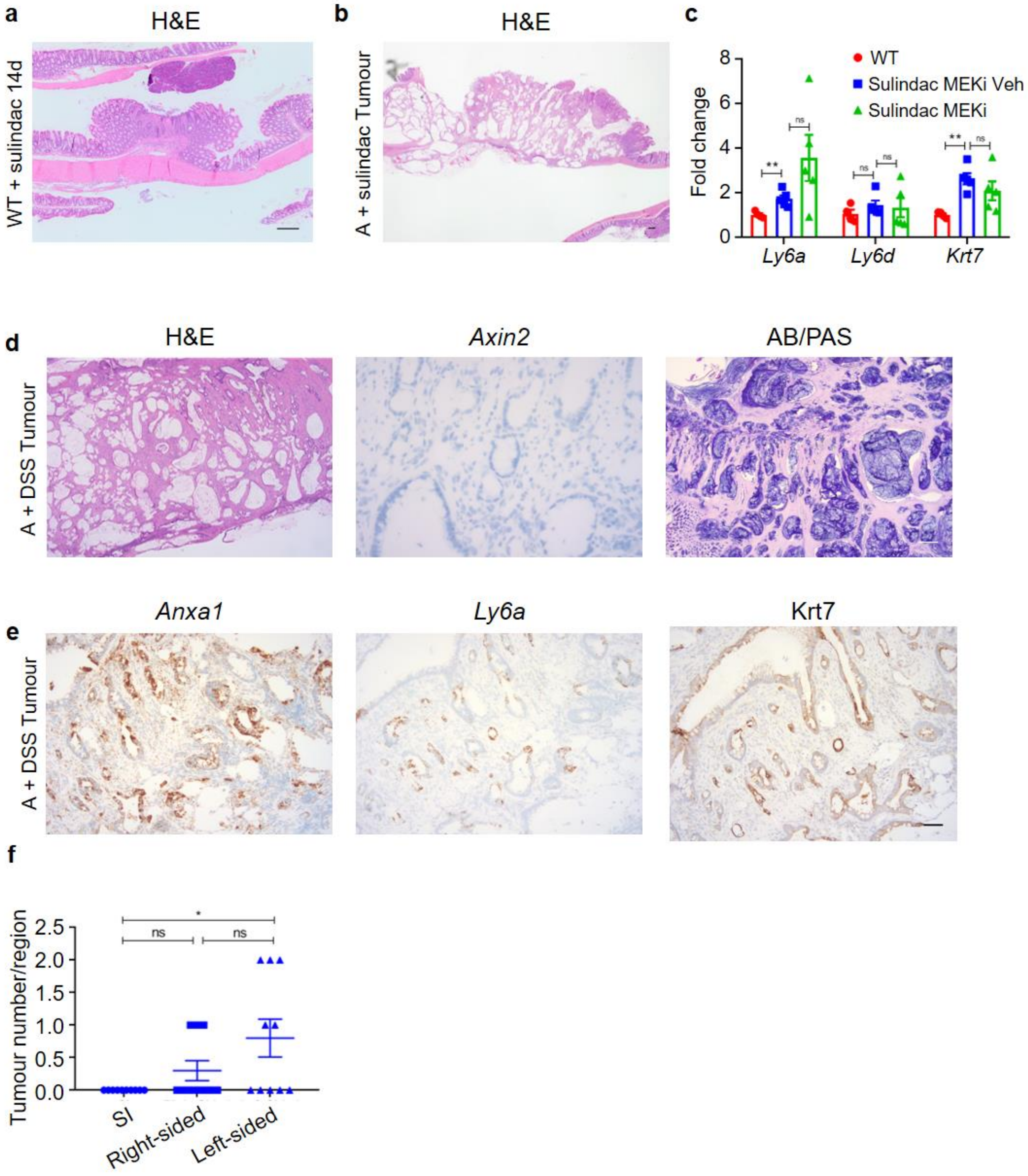

Supplementary Fig. 7: Loss of epithelial TGFβ-receptor, in the context of inflammation, allows formation of foetal-like tumours.

a, Representative H&E-staining of the proximal colon from WT mice treated with high-dose sulindac. n=5 mice. Scale bar, 500 µm. **b**, Representative H&E-staining of a proximal colonic adenocarcinoma from a sulindac-treated A mouse (n=5 mice). Scale bar, 1.0 mm. **c**, qRT-PCR of indicated foetal markers in proximal colonic tissue from sulindac-treated WT mice treated with MEKi (AZD6244; n=5) or Vehicle (n=5) compared with WT control counterparts (n=4), 14 days post sulindac-treatment. Fold change is shown relative to untreated WT mice. Mean ± s.e.m. (ns, not significant; ***p*=0.0079; Mann–Whitney *U*-test, two-tailed). **d**, Representative staining of colonic tumours from DSS-treated A mice. H&E (left panel); *Axin2* ISH (middle panel); Alcian Blue/PAS (right panel). n=5 tumours from 5 DSS-treated mice. Scale bar, 500 µm. **e**, Representative ISH of colonic tumours from DSS-treated A mice, stained for *Anxa1*, *Ly6a*, and *Krt7*. n=5 tumours from 5 DSS-treated mice. Scale bar, 500 µm. **f**, Intestinal tumour location scoring of DSS-treated A mice (n=10). Mean ± s.e.m. (ns, not significant; **p*=0.0325, Mann–Whitney *U*-test, two-tailed).

Supplementary Table 1 – Geneset enrichment analyses carried out in this study

**BA tumour vs WT proximal colon**	**Enrichment Score (ES)**	**Normalised Enrichment Score (NES)**	**False Discovery Rate (FDR *q*-value)**	***p*-value**
Mustata Foetal Spheroid Upregulated	0.426	7.600	0.0	0.0
Hallmark Inflammatory Response	0.379	5.500	0.0	0.0
Gregorieff YAP up	0.383	8.096	0.0	0.0
Gregorieff YAP down	-0.250	-4.971	0.0	0.0

**BA d30 vs WT proximal colon**	**Enrichment Score (ES)**	**Normalised Enrichment Score (NES)**	**False Discovery Rate (FDR *q*-value)**	***p*-value**
Mustata Foetal Spheroid Upregulated	0.247	4.389	0.0	0.0
Munoz Lgr5+ Signature	-0.277	-3.805	0.0	0.0
Gregorieff YAP up	0.301	6.394	0.0	0.0
Gregorieff YAP down	-0.221	-0.582	0.0	0.0

**A+Sulindac vs WT proximal colon**	**Enrichment Score (ES)**	**Normalised Enrichment Score (NES)**	**False Discovery Rate (FDR *q*-value)**	***p*-value**
Mustata Foetal Spheroid Upregulated	0.191	3.432	0.0	0.0
Munoz Lgr5+ Signature	-0.143	-1.948	0.020	0.008

Supplementary Table 2 – qPCR primers pairs used in this study

Gene	Forward (5'-3')	Reverse (5'-3')
Gapdh	GAAGGCCGGGGCCCACTTGA	CTGGGTGGCAGTGATGGCATGG
Ly6a	AGGAGGCAGCAGTTATTGTGG	CGTTGACCTTAGTACCCAGGA
Ly6d	GCCTGGGCACTTCGATGTC	TGAGTTTGCACACTCTTTCCTC
Krt7	AGGAGATCAACCGACGCAC	GTCTCGTGAAGGGTCTTGAGG
Dusp6	ATAGATACGCTCAGACCCGTG	ATCAGCAGAAGCCGTTCGTT
Lgr5	GAGTCAACCCAAGCCTTAGTATCC	CATGGGACAAATGCAACTGAAG
