# Supplementary material for: Oncogenic BRAF, unrestrained by TGFβ-receptor signalling, drives right-sided colonic tumorigenesis
Source: Nat Commun. 2021 Jun 8;12:3464. doi: 10.1038/s41467-021-23717-5 (PMC8187652; doi:10.1038/s41467-021-23717-5)
Supplement: Supplementary file 2 — Reporting Summary [file 41467_2021_23717_MOESM2_ESM.pdf]

nature research

Corresponding author(s): Owen Sansom

Last updated by author(s): Apr 22, 2021

Reporting Summary

Nature Research wishes to improve the reproducibility of the work that we publish. This form provides structure for consistency and transparency in reporting. For further information on Nature Research policies, see our Editorial Policies and the Editorial Policy Checklist.

Statistics

For all statistical analyses, confirm that the following items are present in the figure legend, table legend, main text, or Methods section.

n/a	Confirmed	
☐	☒	The exact sample size (n) for each experimental group/condition, given as a discrete number and unit of measurement
☐	☒	A statement on whether measurements were taken from distinct samples or whether the same sample was measured repeatedly
☐	☒	The statistical test(s) used AND whether they are one- or two-sided<br>*Only common tests should be described solely by name; describe more complex techniques in the Methods section.*
☒	☐	A description of all covariates tested
☒	☐	A description of any assumptions or corrections, such as tests of normality and adjustment for multiple comparisons
☐	☒	A full description of the statistical parameters including central tendency (e.g. means) or other basic estimates (e.g. regression coefficient) AND variation (e.g. standard deviation) or associated estimates of uncertainty (e.g. confidence intervals)
☐	☒	For null hypothesis testing, the test statistic (e.g. F, t, r) with confidence intervals, effect sizes, degrees of freedom and P value noted<br>*Give P values as exact values whenever suitable.*
☒	☐	For Bayesian analysis, information on the choice of priors and Markov chain Monte Carlo settings
☒	☐	For hierarchical and complex designs, identification of the appropriate level for tests and full reporting of outcomes
☐	☒	Estimates of effect sizes (e.g. Cohen's d, Pearson's r), indicating how they were calculated

Our web collection on statistics for biologists contains articles on many of the points above.

Software and code

Policy information about availability of computer code

Data collection	Applied Biosystems StepOnePlus PCR system using StepOne software version 2.3 (qPCR).
Data analysis	Data analysis was undertaken using -<br>Microsoft Excel 2016<br>Graphpad Prism version 8.02<br>HALO V2.0.1145 (Indica Labs)<br>FastQC v0.11.8<br>TrimGalore! v0.6.4<br>HISAT2 v2.1.0<br>FeatureCounts v1.6.4<br>DESeq2 v1.22.2<br>edgeR v3.28.1<br>Limma<br>R v3.6.0

For manuscripts utilizing custom algorithms or software that are central to the research but not yet described in published literature, software must be made available to editors and reviewers. We strongly encourage code deposition in a community repository (e.g. GitHub). See the Nature Research guidelines for submitting code & software for further information.

Data

Policy information about availability of data

All manuscripts must include a data availability statement. This statement should provide the following information, where applicable:

- Accession codes, unique identifiers, or web links for publicly available datasets
- A list of figures that have associated raw data
- A description of any restrictions on data availability

Data availability - All data relevant to this study are available from the authors at reasonable request. The RNAseq datasets that support the findings described here have been deposited at NCBI Gene Expression Omnibus (https://www.ncbi.nlm.nih.gov/geo) with the study accession code GSE168478. All source data are available as a Source Data file. The remaining data are available within the Article, Supplementary Information or available from the authors upon request.

Field-specific reporting

Please select the one below that is the best fit for your research. If you are not sure, read the appropriate sections before making your selection.

☒ Life sciences ☐ Behavioural & social sciences ☐ Ecological, evolutionary & environmental sciences

For a reference copy of the document with all sections, see nature.com/documents/nr-reporting-summary-flat.pdf

Life sciences study design

All studies must disclose on these points even when the disclosure is negative.

Sample size	For all in vivo experiments, power analyses were carried out to determine cohort sizes based upon effect size and SD derived from unpublished experiments in similar GA models previously carried out within the lab, and from early pilot studies which were carried out within experimental and control cohorts. Power analyses were carried out using the G* power software package 3.1.9.4 (HHU Dusseldorf), typically defining alpha=0.05 and beta=0.2. Animal studies were also carried out respecting the limited use of animals in line with the 3R system: Replacement, Reduction, Refinement. For all other experiments, sample sizes were not statistically pre-determined, but were based upon results from prior experiments in these and related model systems.
Data exclusions	No data were excluded, unless mentioned otherwise.
Replication	For all in vivo and ex vivo experiments carried out, individual animals of control and experimental cohorts are biologically unique - here replicate data represents analysis of data/samples from independent replicate animals and is denoted by "n". All experiments were repeated on at least three independent occasions using the same experimental approach, and all attempts at replication were successful.
Randomization	To minimise genetic variability, all experimental and control animals were generated on inbred genetic backgrounds, or where that was not possible, were generated from individual breeding colonies. Where possible, control and experimental animals were co-housed independent of genotype and cohorts were comprised of a balance of both male and female animals. in order to reduce the impact of covariates such as gender or housing, animals were recruited to treatment groups in a partially randomised manner while taking these factors into account.
Blinding	For animal welfare reasons, researchers were not blinded to genotype during study and data collection. The investigator(s) were blinded to genotype or treatment during data analysis.

Reporting for specific materials, systems and methods

We require information from authors about some types of materials, experimental systems and methods used in many studies. Here, indicate whether each material, system or method listed is relevant to your study. If you are not sure if a list item applies to your research, read the appropriate section before selecting a response.

Materials & experimental systems

n/a	Involved in the study	
☐	☒	Antibodies
☐	☒	Eukaryotic cell lines
☒	☐	Palaeontology and archaeology
☐	☒	Animals and other organisms
☒	☐	Human research participants
☒	☐	Clinical data
☒	☐	Dual use research of concern

Methods

n/a	Involved in the study	
☒	☐	ChIP-seq
☒	☐	Flow cytometry
☒	☐	MRI-based neuroimaging

Antibodies

Antibodies used	Antibodies, along with their catalog numbers are included within the manuscript -<br>Cytokeratin-7 (Abcam, ab9021)<br>Cleaved-Caspase-3 (CST, #9661)<br>BrdU (BD Biosciences, #347580)<br>RFP (CST, #2555)<br>β-catenin (BD Biosciences, #610154)<br>SCA1/LY6A (R&D Biosystems, #177228)<br>YAP (CST, #4912)
Validation	Antibodies, along with their catalog numbers are included within the manuscript -<br>1.Cytokeratin-7 (Abcam, ab9021); validated by immunohistochemistry (including mouse). Species reactivity: Human, Pig<br>2. Cleaved-Caspase-3 (CST, #9661); validated by western blot, immunoprecipitation, immunohistochemistry, immunofluorescence and flow cytometry. Species reactivity: Human, Mouse, Rat, Monkey<br>3. BrdU (BD Biosciences #347580, 1/200 dilution); validated by flow cytometry. Species Reactivity: Mouse<br>4. RFP (CST, #2555); validated by western blotting and immunohisstochemistry. Species Reactivity: NA - exogenous expression only<br>5. β-catenin (BD Biosciences, #610154); validated by western blot, immunofluorescence, immunohistochemistry and immunoprecipitation. Species reactivity: Human, Mouse, Rat, Dog, Chicken<br>6. SCA1/LY6A (R&D Biosystems, #177228); validated by ELISA, immunocytochemistry and flow cytometry. Species Reactivity: Mouse<br>7. YAP (CST, #4912); validated by western blot and immunoprecipitation. Species Reactivity: Human, Rat, Mouse, Monkey

Eukaryotic cell lines

Policy information about cell lines

Cell line source(s)	3D organoid lines from mouse intestinal crypts were derived at the Cancer Research UK Beatson Institute
Authentication	Organoid lines were authenticated by PCR genotyping
Mycoplasma contamination	All cell lines are routinely tested for mycoplasma and it is considered that the lines used in the study are mycoplasma free.
Commonly misidentified lines<br>(See ICLAC register)	No commonly misidentified lines were used in the study

Animals and other organisms

Policy information about studies involving animals; ARRIVE guidelines recommended for reporting animal research

Laboratory animals	Adult male and female genetically engineered mice of a C57BL/6J background were used in this study. Animals entered study at between 6-12 weeks of age, and only once they had reached a minimum body weight of 20g. Mice were assessed for symptoms of ill health at least 3 times per week, and humanely culled at upon reaching a clinical endpoint in line with UK Home Office regulations. Animals were housed in conventional caging, with environmental enrichment on a 12 hour light-dark cycle in a temperature and humidity controlled environment, with access to food and water ad libitum.
Wild animals	No wild animals were used in the study
Field-collected samples	No field-collected samples were used in this study.
Ethics oversight	All animal experiments were performed in accordance with UK Home Office regulations (Project licence 70/8646), with adherence to the ARRIVE guidelines, and were reviewed and approved by the Animal Welfare and Ethical Review Board (AWERB) of the University of Glasgow.

Note that full information on the approval of the study protocol must also be provided in the manuscript.
